# Association of *NLRP3* rs10754558 polymorphism with inflammasome-related cytokine responses in chronic spontaneous urticaria

**DOI:** 10.3389/fimmu.2026.1804228

**Published:** 2026-05-08

**Authors:** Noha M. Hammad, Arwa O. Ali, Hagar Nofal, Noura Mostafa Mohamed, Tarek Gheith

**Affiliations:** 1 Department of Medical Microbiology and Immunology, Faculty of Medicine, Zagazig University, Zagazig, Egypt; 2 Al Jouf Regional Laboratory, Shared Services, Ministry of Health, Sakaka, Saudi Arabia; 3 Department of Dermatology, Venereology and Andrology, Faculty of Medicine, Zagazig University, Zagazig, Egypt; 4 Department of Basic Sciences, College of Medicine, Princess Nourah bint Abdulrahman University, Riyadh, Saudi Arabia

**Keywords:** chronic spontaneous urticaria, IL-18, IL-1β, innate immunity, NLRP3 inflammasome, NLRP3 rs10754558 polymorphism

## Abstract

**Background:**

Chronic spontaneous urticaria (CSU) is a clinically heterogeneous inflammatory condition affecting the skin, in which mechanisms beyond histamine-mediated mast cell activation are increasingly recognized. Dysregulation of innate immune pathways, including inflammasome signaling, may contribute to disease pathogenesis. Within the inflammasome family, NOD-like receptor pyrin domain–containing protein 3 (NLRP3) rs10754558 polymorphism has been implicated in altered cytokine responses in other inflammatory conditions. This study aimed to investigate the association between *NLRP3* rs10754558 polymorphism and CSU susceptibility, and to examine its relationship with inflammasome-related cytokine responses (interleukin-1β [IL-1β] and IL-18).

**Methods:**

Fifty-six patients with CSU and 56 age- and sex-matched healthy controls were recruited. Genotyping of *NLRP3* rs10754558 was performed. Whole blood cultures were stimulated with lipopolysaccharide (LPS) to assess IL-1β and IL-18 production. Clinical and laboratory parameters, including the urticaria activity score (UAS7), autologous serum skin test (ASST) reactivity, erythrocyte sedimentation rate (ESR), and total serum IgE, were also evaluated.

**Results:**

The C allele of *NLRP3* rs10754558 was significantly associated with increased CSU risk across multiple genetic models. CSU patients showed higher LPS-stimulated IL-1β and IL-18 levels than controls, with C/C genotype carriers exhibiting the highest responses. IL-1β and IL-18 showed a modest positive correlation (r = 0.37), and cytokine responses showed limited associations with conventional severity measures.

**Conclusions:**

The *NLRP3* rs10754558 C allele is associated with heightened systemic inflammasome-related cytokine responses in CSU. These findings are associative and indicate a potential link between genetic variation and innate immune responsiveness, while direct mechanistic confirmation of NLRP3 inflammasome activation in CSU remains to be investigated.

## 1 Introduction

Chronic spontaneous urticaria (CSU) is a heterogeneous inflammatory skin condition characterized by the spontaneous recurrence of pruritic wheals, angioedema, or both for more than six weeks in the absence of an identifiable external trigger (1). Although mast cell (MC)–mediated histamine release represents a central effector mechanism in CSU (2), accumulating evidence suggests that dysregulation of innate immune pathways contributes to disease heterogeneity and persistence (3). In this context, inflammasome signaling has emerged as a biologically plausible link between genetic susceptibility and downstream inflammatory responses in CSU (4).

Inflammasomes are cytosolic multiprotein complexes that activate caspase-1, thereby promoting the conversion of pro–interleukin-1β (IL-1β) and pro–interleukin-18 (IL-18) into their active forms. These cytokines contribute to a wide range of inflammatory and autoimmune skin disorders, including urticaria (4–6). Among the various inflammasome complexes, NOD-like receptor pyrin domain–containing protein 3 (NLRP3) is the most extensively studied and has been implicated in numerous autoinflammatory and autoimmune conditions (2).

Previous studies have reported that total IL-18 levels correlate with clinical severity in CSU, as assessed by the autologous serum skin test (ASST). However, Puxeddu et al. (2013) demonstrated significantly elevated levels of free IL-18 and IL-18 binding protein in CSU patients, irrespective of ASST status (7). In addition, MCs, which play a central role in CSU pathogenesis, are an important source of IL-1β. This cytokine promotes the expression of inflammatory mediators such as prostaglandins and platelet-activating factor, and facilitates neutrophil recruitment and extravasation, thereby contributing to wheal formation. Despite these findings, the precise roles of MC–derived IL-1β and IL-18 in CSU, particularly in the context of innate and adaptive immune responses, remain to be fully elucidated (6).

The functional relevance of genetic variation within the *NLRP3* gene has gained increasing attention. The *NLRP3* rs10754558 polymorphism, located in the 3′ untranslated region, influences NLRP3 mRNA stability and has been associated with altered cytokine production (8). This variant has been linked to susceptibility to a broad spectrum of conditions, including infectious diseases (9), neoplastic processes (10), hematological disorders (11), inflammatory and autoimmune diseases (12). However, its contribution to CSU remains incompletely characterized (13).

Given the emerging role of inflammasome signalling, this case–control study aimed to investigate the association between the *NLRP3* rs10754558 polymorphism and susceptibility to CSU, and to evaluate inflammasome-related cytokine responses (IL-1β and IL-18) following lipopolysaccharide (LPS) stimulation in whole blood cultures, as IL-1β and IL-18 are key downstream mediators of NLRP3 inflammasome activation, the primary pathway under investigation. In addition, we examined the relationships between NLRP3 genotypes, cytokine responses, and key clinical and laboratory features of CSU to better define the immunogenetic landscape of this condition.

## 2 Methods

### 2.1 Sample size calculation

Sample size was determined using previously reported IL-18 measurements by Abdel-Bary et al. (2022) (14). Assuming mean IL-18 levels of 115.6 ± 121.1 pg/mL in the control group and 214.9 ± 167.3 pg/mL in the CSU group, at least 46 participants per group were needed to ensure 80% power with a 95% confidence level. To accommodate biological variability, potential dropouts, and the application of non-parametric tests, the cohort was expanded to 56 subjects per group. Sample size calculations were conducted using OpenEpi software.

### 2.2 Study design

A case–control study was conducted from December 2022 to December 2025, including 56 patients with CSU and 56 healthy volunteers matched by age and sex. Patients were recruited from the Allergy and Immunology Unit and the Dermatology Outpatient Clinic at Zagazig University Hospitals, Zagazig University. Immunological assays and cell culture experiments were performed at the Immunology Research Laboratory (IRL), Faculty of Medicine, Zagazig University, within the Medical Microbiology and Immunology Department, while molecular analyses were conducted at the Molecular Biology Unit of the Scientific and Medical Research Center of Zagazig University (ZSMRC). The study protocol was approved by the Institutional Review Board of Faculty of Medicine, Zagazig University (ZU-IRB# 9686/17-8-2022).

### 2.3 Inclusion and exclusion criteria

Patients presenting with chronic urticaria, characterized by wheals with or without angioedema lasting longer than six weeks, were eligible for inclusion. Exclusion criteria comprised identifiable causes of urticaria, other pruritic or autoimmune skin diseases, recent use of immune-modifying therapies or immunoglobulin/plasmapheresis, significant comorbid conditions affecting interpretation of results, and refusal to participate.

Healthy controls were age- and sex-matched individuals with no history of chronic urticaria, angioedema, autoimmune, or inflammatory diseases. Controls were not taking medications affecting immune function, including corticosteroids, immunosuppressants, or antihistamines, and had no acute infections within the preceding four weeks. All participants provided written informed consent prior to blood sampling.

### 2.4 Urticaria activity score

Urticaria severity was evaluated using the UAS7 index, which employs a four-point scale (0–3) for both wheals and pruritus. Daily scores for wheals and pruritus were combined (total 0–6) and recorded over seven consecutive days (15). UAS7 was calculated in each patient by adding the sum of daily score to a total of 42. Patients with UAS7 ≤ 6 were considered well controlled, those with UAS7 score 7–15 were considered mild symptomatic, while those with UAS7 score of 16–27 or 28–42 were considered moderate and severe urticaria, respectively.

### 2.5 Body mass index

In CSU patients, BMI was adjusted by normalizing body weight in kilograms to stature expressed in meters squared (kg/m²) (16).

### 2.6 Blood sampling

Five millilitres of peripheral blood were aseptically drawn from each participant into three vacutainer tubes: lithium heparin for whole blood culture, EDTA for DNA extraction and genotyping, and a serum separator tube. The serum sample was allowed to clot, centrifuged, and the separated serum was used for ASST.

### 2.7 Autologous serum skin test

The ASST was performed by intradermal injection of undiluted patient serum, with normal saline as a negative control and histamine as a positive control, following standard procedures (**Supplementary Figure S1**) (17). Wheal responses were evaluated after 30 minutes, and ASST was deemed positive if the mean wheal diameter of the serum injection site exceeded that of the negative control by ≥ 1.5 mm (17).

### 2.8 Whole blood cell culture assay

According to Rodas et al. (2021), one milliliter of heparinized blood was diluted 1:1 with culture medium (RPMI-1640 Medium, Sigma, St Louis, MO, USA) enriched with penicillin (100 U/mL) and streptomycin (100 µg/mL) (10000 u/mL penicillin and 10000 μg/mL streptomycin, Invitrogen corp./Gibco), then divided into two cultures: one stimulated with LPS (Escherichia coli serotype 0111: B4; Sigma, St Louis, MO, USA) to achieve a concentration of 10 ng/mL while the second culture was maintained as an unstimulated control (18). Following a 24-hour incubation at 37°C in a humidified atmosphere with 5% CO_2_, cultures were centrifuged at 1000 × g for 10 minutes. The supernatants were then collected and stored at −20°C for later cytokine measurements (**Supplementary Figure S2**).

### 2.9 Assessment of IL-1β and IL-18 in supernatants of cultured cells

Concentrations of IL-1β and IL-18 in the collected culture supernatants were determined using sandwich ELISA following the manufacturer’s instructions (Human Interleukin 1β and Human Interleukin 18 ELISA Kits, Cloud-Clone Corp., USA, Cat No. E-00860hu and E-00857hu, respectively).

### 2.10 NLRP3 rs10754558 polymorphism genotyping

DNA was extracted from participants’ whole blood samples using the GeneJET™ Whole Blood Genomic DNA Purification Mini Kit (Thermo Fisher, USA) following the manufacturer’s protocol. DNA yield and purity were measured to ensure suitability for downstream genotyping by UV/Vis spectrophotometry (19), and samples were stored at −20°C until analysis.

Allelic discrimination of the *NLRP3* rs10754558 polymorphism was performed using a TaqMan^®^ 5′-nuclease real-time PCR assay (ThermoFisher, USA) on a StepOne™ Real-Time PCR system (Applied Biosystems, USA). The assay employed sequence-specific forward and reverse primers to amplify the rs10754558 region: (GACAATGACAGCATCGGGTGTTGTT[C/G]TCATCACAGCGCCTCAGTTAGAGGA), together with VIC^®^- and FAM™-labeled minor groove binder (MGB) probes specific for the C and G alleles, respectively. PCR reactions (20 µL) were prepared with 1× TaqMan Genotyping Master Mix, 1× SNP Genotyping Assay, and 1–20 ng of genomic DNA. Thermal cycling consisted of an initial enzyme activation at 95°C for 10 minutes, followed by 40 cycles of 95°C for 15 seconds and 60°C for 1 minute for annealing/extension. Genotypes were determined by endpoint fluorescence analysis, where VIC fluorescence indicated homozygosity for the C allele, FAM fluorescence indicated homozygosity for the G allele, and combined fluorescence indicated C/G heterozygosity (**Supplementary Figures S3–S7**).

### 2.11 Other laboratory investigations

As part of the study protocol, all CSU patients underwent routine laboratory investigations, including CBC, ESR, CRP, thyroid antibodies, and serum total IgE.

### 2.12 Statistical analysis

Data were processed using SPSS v24.0, with graphs generated via SRPLOT. Continuous variables are expressed as mean ± standard deviation (SD), while categorical variables are reported as counts and percentages. Normally distributed data were analyzed using parametric tests (t-tests and ANOVA), while non-normally distributed data were analyzed with nonparametric tests (Mann–Whitney U and Kruskal–Wallis). Categorical variables were compared using Pearson’s chi-square or Fisher’s exact tests, as appropriate. Genotype and allele frequencies were calculated by direct counting., and Hardy–Weinberg equilibrium was evaluated using χ² goodness-of-fit. Correlations were assessed using Pearson or Spearman correlation coefficients as appropriate. All analyses were two-tailed, and p ≤ 0.05 was considered statistically significant.

## 3 Results

### 3.1 Demographic and clinical characteristics of participants

The present study enrolled 56 CSU patients and 56 age- and sex- matched healthy controls. The mean age of CSU patients was 33.4 ± 7.4 years, compared with 35.8 ± 8.3 years in the control group, with no statistically significant difference between groups (*p* = 0.1). Female participants predominated in both the CSU (67.9%) and control (76.8%) groups, with no significant difference in sex distribution (*p* = 0.3). Baseline, clinical, and laboratory features of study participants are presented in **Table 1**.

**Table 1:** Baseline characteristics of study participants and clinical/laboratory features CSU patients (N = 112).

Variable	CSU patients	Controls	Test of significance	P-value
	N = (56)	N = (56)		
Age (Years)			Independent sample *t*-test	0.1
Mean ± SD	33.4 ± 7.4	35.8 ± 8.3		
Medium (min-mix)	33.5 (17.0-54.0)	37 (20-59)		
Sex			Chi square test	0.3
Female	38 (67.9)	43 (76.8%)		
Male	18 (32.1)	13 (23.2%)		
BMI		NA	–	–
Mean ± SD	31.6 ± 5.0			
Medium (min-mix)	32.0 (20.0 - 44.0)			
ASST		NA	–	–
Negative	16 (28.6%)			
Positive	40 (71.4%)			
UAS7		NA	–	–
Mean ± SD	17.1 ± 5.9			
Medium (min-mix)	15 (10-42)			
ESR		NA	–	–
Normal	28 (50.0%)			
Elevated	28 (50.0%)			
CRP		NA	–	–
Negative	55 (98.2%)			
Positive	1 (1.8%)			
Thyroid antibodies		NA	–	–
Negative	55 (98.2%)			
Positive	1 (1.8%)			
Eosinophil count		NA	–	–
Normal	56 (100%)			
Basophil count		NA	–	–
Normal	56 (100%)			
Total serum IgE (IU/mL)		NA	–	–
Mean ± SD	187.3 ± 100.3			
Medium (min-mix)	172.0 (73.0 - 443.0)			

NA, Non-applicable.

**Figure 1:**
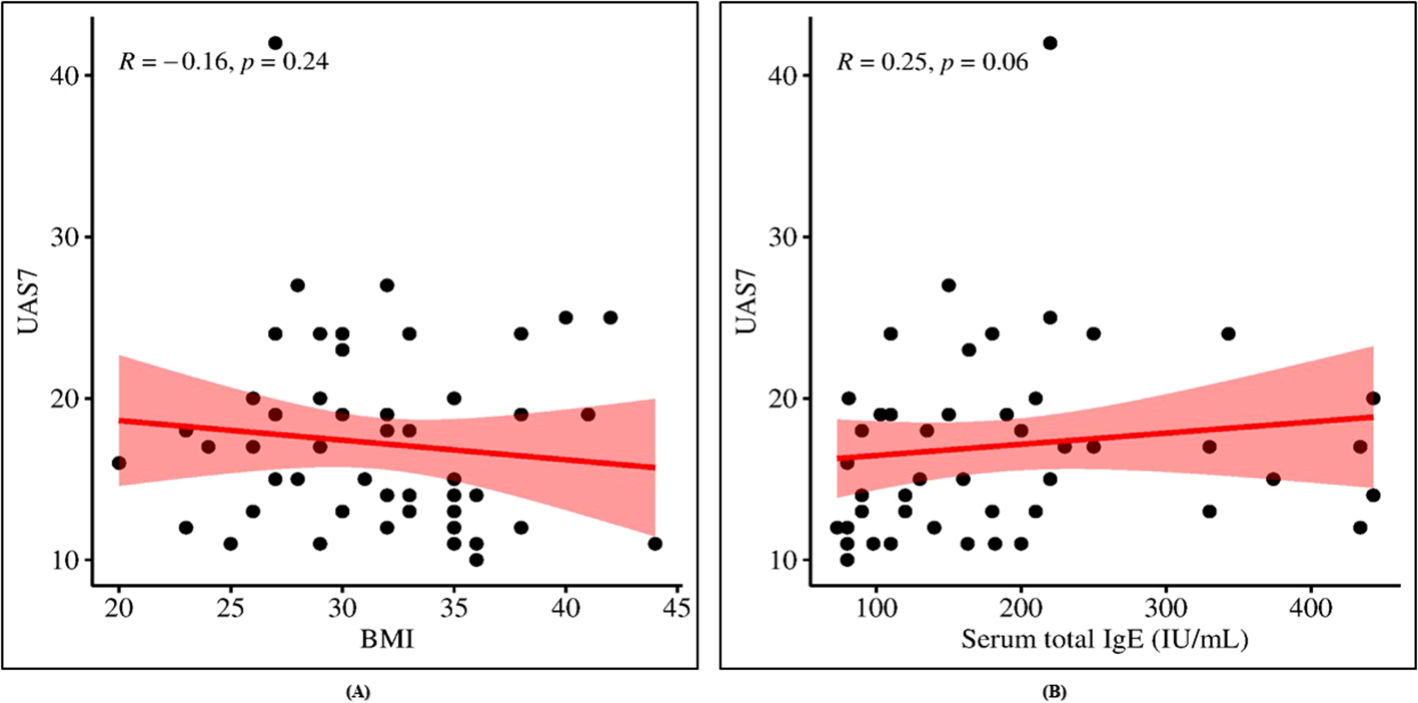
Scatter plots showing non- significant correlation of UAS7 with **(A)** BMI and **(B)** total IgE serum level. Data were analyzed by Spearman correlation.

**Figure 2:**
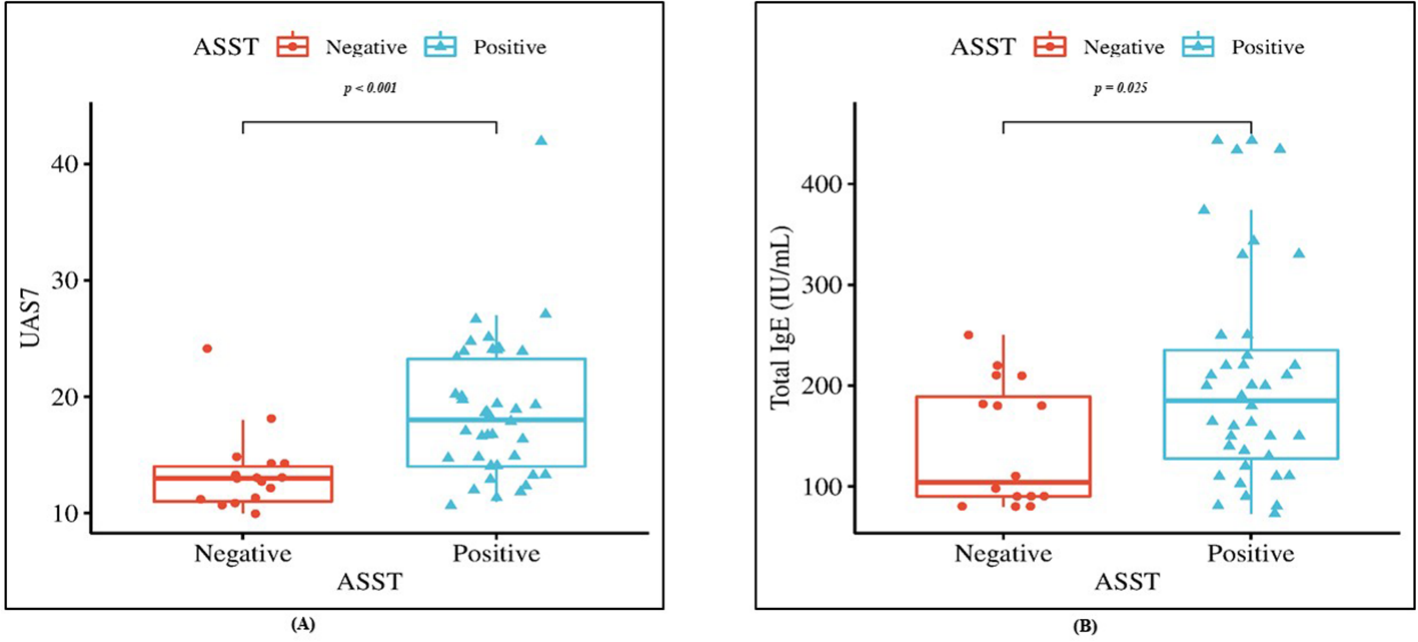
Box plot showing **(A)** significant higher UAS7 and **(B)** total IgE serum level in ASST positive CSU patients than ASST negative patients. Data were analyzed by t test and Mann-Whitney U test, respectively.

UAS7 scores were not significantly correlated with BMI or total serum IgE concentrations (*p* = 0.20 and *p* = 0.06, respectively; **Figures 1A, B**). Stratification of CSU patients by ASST status revealed significantly higher UAS7 scores in ASST-positive patients compared with ASST-negative patients (*p* < 0.001; **Figure 2A**). Furthermore, total serum IgE levels were significantly elevated in the ASST-positive group (*p* = 0.025; **Figure 2B**).

### 3.2 Association of *NLRP3* rs10754558 polymorphism with CSU Susceptibility

**Figure 3:**
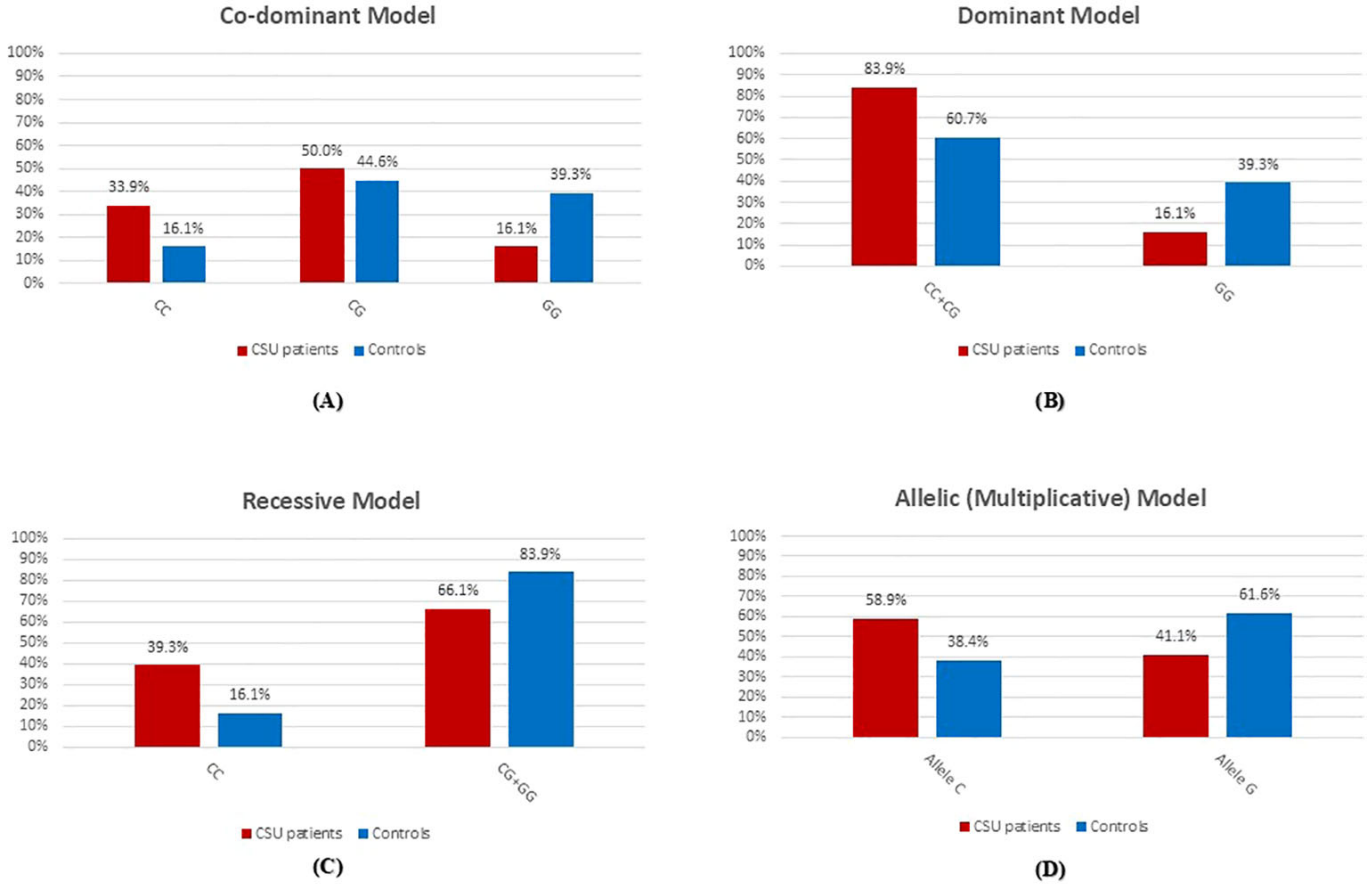
Genotype and allele distributions of *NLRP3* rs10754558 in CSU patients and healthy controls across different genetic models. Bar charts show the distribution of *NLRP3* rs10754558 genotypes and alleles in CSU patients (red bars, n = 56) and healthy controls (blue bars, n = 56). **(A)** Co-dominant model: C/C, C/G, and G/G genotypes. **(B)** Dominant model: C/C + C/G versus G/G genotypes. **(C)** Recessive model: C/C versus C/G + G/G genotypes. **(D)** Allelic (multiplicative) model: C versus G alleles.

**Table 2** shows a significant association between the *NLRP3* rs10754558 polymorphism and CSU across all tested genetic models, including codominant, dominant, recessive, and multiplicative models (*p* = 0.009, 0.006, 0.03, and 0.002, respectively). Under the codominant model, individuals carrying the C/C genotype had a 5.2-fold increased risk of CSU compared with G/G carriers and a 1.9-fold higher risk compared with C/G carriers. In the dominant model, carriers of the C allele (C/C or C/G) exhibited a 3.4-fold higher risk of CSU relative to G/G genotype carriers. Under the recessive model, the C/C genotype was associated with a 2.7-fold increased risk of CSU compared with C/G and G/G genotypes. Similarly, the multiplicative model indicated that carriage of the minor C allele conferred a 2.3-fold higher risk of developing CSU compared with the major G allele. Genotype and allele distributions of *NLRP3* rs10754558 across different genetic models are shown in **Figure 3**.

**Table 2:** *NLRP3* rs10754558 genotypes distribution in CSU patients and controls (N = 112).

Model	Genotype	CSU patients	Controls	Test of significance	*P-value*	OR
		N = (56)	N = (56)			(95% CI)
		n (%)	n (%)			
Co-dominant	C/C	19 (33.9)	9 (16.1)	Chi square test	0.009*	1
	C/G	28 (50.0)	25 (44.6)			1.9 (0.7-4.9)
	G/G	9 (16.1)	22 (39.3)			5.2 (1.7-15.6)
Dominant	C/C-C/G	47 (83.9)	34 (60.7)		0.006*	1
	G/G	9 (16.1)	22 (39.3)			3.4 (1.4-8.2)
Recessive	C/C	19 (33.9)	9 (16.1)		0.03*	1
	C/G-G/G	37 (66.1)	47 (83.9)			2.7 (1.1-6.6)
Multiplicative^♠^	C	66 (58.9)	43 (38.4)		0.002*	1
	G	46 (41.1)	69 (61.6)			2.3 (1.3-3.9)

*Significant difference.

^♠^ N=224.

Following the observed association between *NLRP3* rs10754558 and CSU susceptibility, genotype and allele distributions were assessed for conformity with Hardy–Weinberg equilibrium. As shown in **Table 3**, no significant deviations from Hardy–Weinberg equilibrium were observed in the overall study population or when CSU patients and controls were analyzed separately (*p* = 0.57, 1.00, and 0.78, respectively).

**Table 3:** Hardy-Weinberg equilibrium.

	N_G/G_	N_C/G_	N_C/C_	N_G_	N_C_	*P-value*
All participants	31	53	28	115	109	0.57
CSU patients	9	28	19	46	66	1
Controls	22	25	9	69	43	0.78

NC/C; observed frequency of genotype C/C, NC/G; observed frequency of genotype C/G, NG/G; observed frequency of genotype G/G, NC; observed frequency of allele C, NG; observed frequency of allele G.

Test of significance chi square goodness of fit test.

### 3.3 LPS-stimulated inflammasome-related cytokine responses

**Figure 4:**
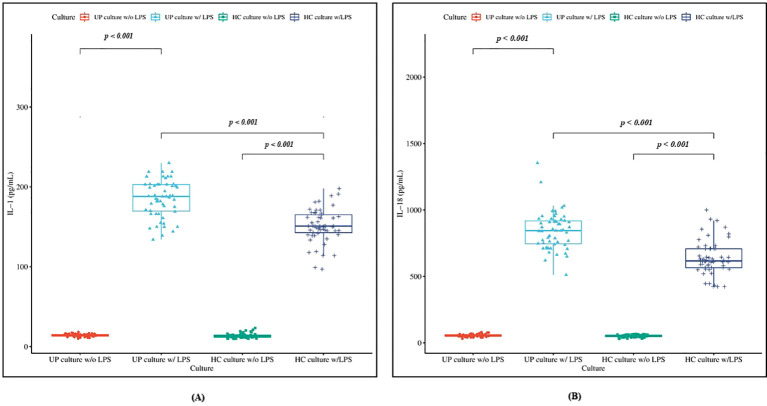
Box plot illustrating **(A)** IL-1β production in LPS unstimulated and stimulated cultures in CSU patients and controls. UP; urticaria patients, w/o LPS; without LPS, w/LPS; with LPS, HC; healthy controls. Data were analyzed by Independent sample t test. **(B)** IL-18 production in LPS unstimulated and stimulated cultures in CSU patients and controls. UP; urticaria patients, w/o LPS; without LPS, w/LPS; with LPS, HC; healthy controls. Data were analyzed by t test.

To assess inflammasome-related cytokine responses, IL-1β and IL-18 production were measured in unstimulated (LPS^-^) and LPS-stimulated (LPS^+^) whole blood cultures (**Figure 4**). In LPS^-^ cultures, IL-1β levels did not differ significantly between CSU patients and controls (14.2 ± 1.7 pg/mL vs. 13.6 ± 2.9 pg/mL, *p* = 0.15). In LPS^+^ cultures, IL-1β levels were higher in CSU patients compared with controls (185.7 ± 23.2 pg/mL vs. 151.4 ± 20.7 pg/mL, *p* < 0.001). Moreover, IL-1β production was greater in LPS^+^ than LPS^-^ cultures in both CSU patients and controls (*p* < 0.001 for each) (**Figure 4A**). Similarly, IL-18 levels in LPS^-^ cultures were comparable between CSU patients and controls (55.1 ± 10.2 pg/mL vs. 51.9 ± 8.7 pg/mL, *p* = 0.08), whereas LPS^+^ cultures showed higher IL-18 levels in CSU patients relative to controls (843.1 ± 140.4 pg/mL vs 640.4 ± 128.0 pg/mL, *p* < 0.001). IL-18 concentrations were also greater in LPS^+^ than in LPS^-^ cultures in both groups (*p* < 0.001 for each) (**Figure 4B**).

### 3.4 Correlations between clinical/laboratory parameters and inflammasome -related cytokine responses

The functional and clinical impact of the *NLRP3* rs10754558 polymorphism in CSU patients was evaluated under codominant, dominant, and recessive genetic models (**Table 4**). In the codominant model (C/C, C/G, G/G), IL-1β production differed significantly across genotypes (overall *p* = 0.002), with higher levels in C/C carriers compared with C/G (*p* = 0.005) and G/G carriers (*p* = 0.002). Similarly, IL-18 levels were highest in C/C carriers, followed by C/G and G/G carriers (overall *p* < 0.001; pairwise *p* < 0.001 for both), while UAS7 scores also varied by genotype (*p* = 0.009), being higher in C/C and C/G carriers than in G/G carriers (*p* = 0.006 and 0.034, respectively). Total serum IgE, ASST reactivity, and ESR did not differ significantly among codominant genotypes (*p* = 0.07, 0.8, and 0.5, respectively).

**Table 4:** Distribution of immunological and disease activity parameters across *NLRP3* rs10754558 genetic models.

Genetic model^♠^	LPS induced IL-1β (pg/mL)	LPS induced IL-18 (pg/mL)	+ve ASST reactivity	UAS7	↑ESR	Total IgE (IU/mL)
	Mean ± SD	Mean ± SD	n (%)	Mean ± SD	n (%)	Mean ± SD
Co-dominant						
C/C (n=19)	199.4 ± 17.3	963.4 ± 128.2	13 (68.4)	14.8 ± 4.5	9 (47.4)	192.8 ± 107.5
C/G (n=28)	181.1 ± 24.7	788.0 ± 110.6	21 (75.0)	19.5 ± 6.6	16 (57.1)	199.7 ± 96.1
G/G (n=9)	171.0 ± 14.2	760.7 ± 69.3	6 (66.7)	14.3 ± 3.0	3 (33.3)	137.1 ± 92.4
Test of significance	F test	F test	Fisher exact test	Kruskal–Wallis test	Fisher exact test	Kruskal–Wallis test
*P*-value	0.002*	<0.001*	0.8	0.009*	0.5	0.07
*P-value between C/C and C/G*	0.005*	<0.001*		0.006*		
*P-value between C/C and G/G*	0.002*	<0.001*		0.9		
*P-value between C/G and G/G*	0.2	0.5		0.034*		
Dominant						
C/C-C/G (n=47)	188.5 ± 23.6	858.9 ± 145.5	34 (72.2)	17.6 ± 6.2	25 (53.2)	196.9 ± 99.8
G/G (n=9)	171.0 ± 14.2	760.9 ± 69.3	6 (66.7)	14.3 ± 3.0	3 (33.3)	137.1 ± 92.4
Test of significance	*t-*test	*t-*test	Fisher exact test	Mann-Whitney U test	Fisher exact test	Mann-Whitney U test
*P*-value	0.037*	0.054	0.7	0.2	0.2	0.026*
Recessive						
C/C (n=19)	199.4 ± 17.3	178.7 ± 22.9	13 (68.4)	14.8 ± 4.5	9 (47.4)	192.8 ± 107.5
C/G-G/G (n=37)	178.7 ± 22.9	781.4 ± 101.9	27 (73.0)	18.2 ± 6.3	19 (51.4)	184.5 ± 97.8
Test of significance	*t-*test	*t-*test	Chi square test	Mann-Whitney U test	Chi square test	Mann-Whitney U test
*P*-value	0.001*	<0.001*	0.7	0.028*	0.8	0.9

^♠^ Codominant, dominant, and recessive genetic models were analysed to assess different patterns of allele effect: codominant evaluates each genotype separately, dominant compares carriers of at least one minor allele to homozygous major allele carriers, and recessive compares homozygous minor allele carriers to all others.

*Significant difference.

In the dominant model (C/C + C/G vs. G/G), carriers of the C allele exhibited higher IL-1β and total IgE levels compared with G/G carriers (*p* = 0.037 and 0.026, respectively), whereas IL-18, UAS7, ASST reactivity, and ESR did not differ significantly (*p* = 0.054, 0.2, 0.7, and 0.7, respectively).

In the recessive model (C/C vs C/G + G/G), C/C carriers exhibited significantly higher IL-1β and IL-18 production compared with C/G + G/G carriers (*p* = 0.001 and < 0.001, respectively), while UAS7 scores were significantly lower in C/C carriers (*p* = 0.028). Total serum IgE, ASST reactivity, and ESR did not differ significantly between C/C and C/G + G/G groups (*p* = 0.9, 0.7, and 0.7, respectively).

**Figure 5:**
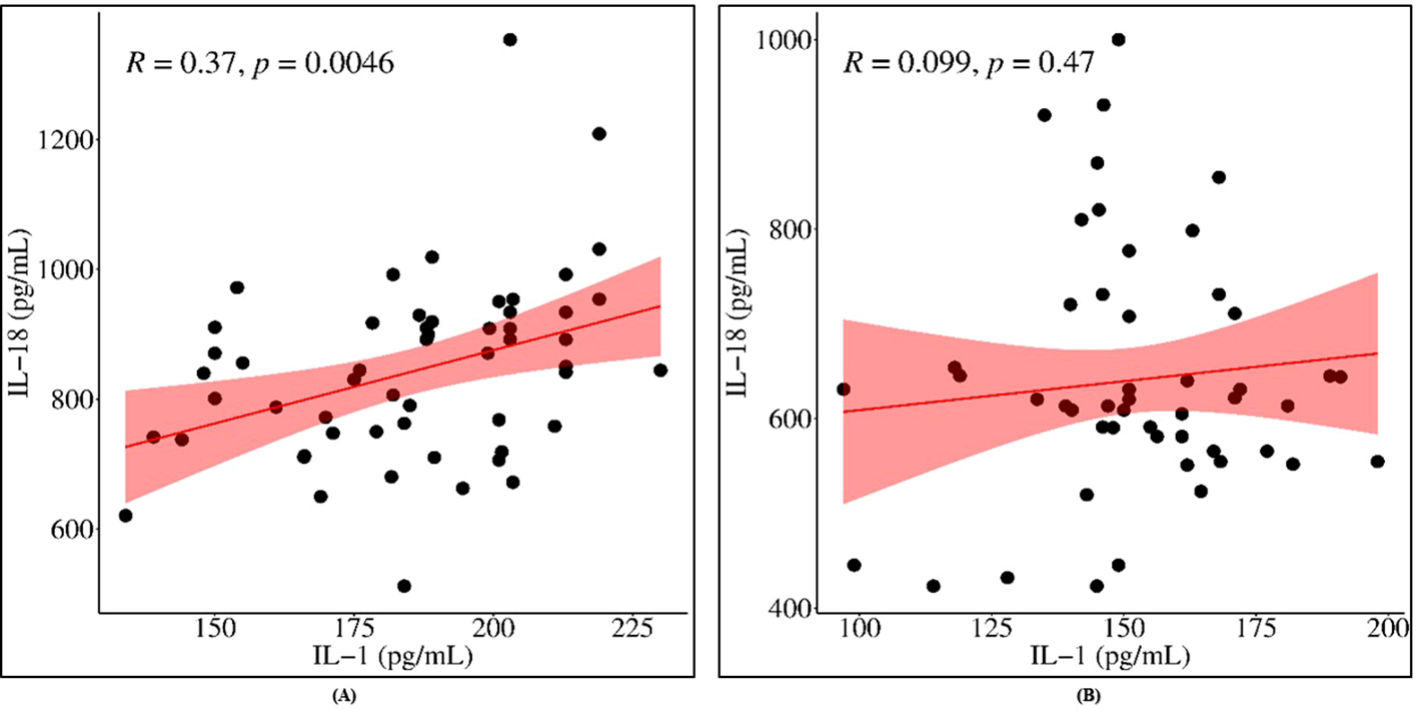
Scatter plots showing non-significant correlations between IL-1β and IL-18 production in LPS challenged cultures in **(A)** CSU patients and **(B)** controls. Data were analyzed by Pearson correlation.

In line with the LPS-stimulated elevations of IL-1β and IL-18 in CSU patients (**Figure 5**), correlation analysis revealed a significant modest positive correlation between IL-1β and IL-18 production in LPS-stimulated cultures from CSU patients (**Figure 5A**, r = 0.37, *p* = 0.005), whereas no such correlation was observed in controls (**Figure 5B**, r = 0.099, *p* = 0.047).

Supplementary analyses explored relationships between clinical and laboratory parameters and their associations with LPS-stimulated inflammasome cytokine responses. These analyses included correlations of UAS7 with BMI and total serum IgE, comparisons by ESR and ASST status, and correlations of LPS-stimulated IL-1β and IL-18 production with age, BMI, UAS7, IgE levels, ESR, and ASST status (**Supplementary Figures S8**-**S11**). No additional statistically significant associations were observed beyond those reported in the main Results.

## 4 Discussion

Current international urticaria guidelines emphasize a structured diagnostic approach for CSU using the 7C concept, which highlights the importance of identifying disease mechanisms, biomarkers, and immune pathways that underlie disease heterogeneity (15). Within this framework, growing attention has focused on innate immune dysregulation as a contributor to CSU pathogenesis. In particular, aberrant activation of inflammasome signaling has emerged as a biologically plausible mechanism linking genetic susceptibility to downstream inflammatory responses in CSU (20).

By integrating genetic association analyses with LPS-stimulated IL-1β and IL-18 measurements, this study examined how the *NLRP3* rs10754558 polymorphism influences both susceptibility to CSU and its downstream immune responses.

The demographic characteristics of the CSU cohort in this study align with the known epidemiology of the disease, which primarily affects adults, with peak onset between the second and fourth decades of life and is associated with considerable impairment in quality of life (21, 22).

Beyond histamine-dependent mechanisms, growing evidence supports a role for histamine-independent inflammatory pathways in CSU. The limited efficacy of antihistamines in a substantial proportion of patients, along with the responsiveness of CAPS-associated urticarial manifestations to IL-1 blockade, highlights the relevance of inflammasome-driven inflammation (2, 23). MCs, as well as macrophages, are capable of NLRP3 inflammasome activation following priming and secondary stimuli, leading to IL-1β release (2, 24). These findings provide a conceptual framework linking innate immune activation to urticarial inflammation.

Within this context, the *NLRP3* rs10754558 polymorphism represents a functionally relevant variant located in the 3′- untranslated region of the gene, where it can influence mRNA stability and downstream cytokine production (8). Although this polymorphism has been studied in several autoimmune and inflammatory diseases with variable results (25–33), only a limited number of investigations have assessed its role in CSU susceptibility. The present study adds to this emerging evidence.

The present study demonstrates a significant association between *NLRP3* rs10754558 and CSU susceptibility across multiple genetic models, with the C allele conferring increased risk. These findings are consistent with reports on systemic lupus erythematosus and type 1 diabetes mellitus (34, 35). However, meta-analyses of autoimmune diseases have described contrasting protective effects of the G allele (8), suggesting that the impact of NLRP3 regulation may be disease-specific or context-dependent. Compared with the C allele, the G allele enhances NLRP3 mRNA stability (~1.3-fold), potentially conferring protection against inflammatory disease, whereas the C allele reduces mRNA stability and may consequently affect IL-1β and IL-18 production (33, 34, 36).

Functionally, our data indicate that *NLRP3* rs10754558 genotypes are associated with differential IL-1β and IL-18 production following LPS-stimulation, with C/C carriers exhibiting the highest cytokine levels. Importantly, IL-1β and IL-18 production was not only increased in CSU patients compared with controls but also showed a modest positive correlation (r = 0.37) in CSU cultures, a pattern not observed in healthy individuals. This correlation should be interpreted with caution, as it does not necessarily indicate direct mechanistic coupling and may reflect partially independent regulatory pathways.

It is important to note that the functional consequences of *NLRP3* rs10754558 polymorphism were evaluated in LPS-stimulated whole blood cultures, which comprise multiple innate immune cell populations, including neutrophils and basophils, and preserve physiological cell–cell interactions. While this approach provides a more integrated view of systemic innate immune responses, it limits precise attribution of cytokine production to specific cell types. Additionally, LPS-stimulation primarily provides a priming signal (signal 1) and does not fully recapitulate canonical NLRP3 inflammasome activation, which typically requires a secondary stimulus such as ATP or nigericin (37). Therefore, the observed increases in IL-1β and IL-18 may reflect enhanced priming, differences in the number or responsiveness of cytokine-producing cells, or broader inflammatory activation rather than definitive inflammasome activation.

Notably, significant IL-1β and IL-18 production was observed following LPS-stimulation alone. This may be explained by the presence of circulating monocytes, which are reported to exhibit constitutive caspase-1 activity and the ability to release endogenous ATP, thereby enabling partial processing of pro–IL-1β and pro–IL-18 even in the absence of a secondary stimulus (38, 39). In contrast, tissue-resident macrophages generally require an additional signal for full inflammasome activation. This difference in responsiveness may contribute to the increased cytokine production observed in CSU and its associated inflammatory milieu. Similar observations have been reported in autoinflammatory syndromes: monocytes from patients with NLRP3 mutations, such as Muckle–Wells syndrome (40), NOMID (41), or hyper-IgD syndrome (42), exhibit increased IL-1β release even in the absence of additional ATP stimulation (40). These findings suggest that NLRP3 variants may lower the threshold for inflammasome activation or enhance constitutive activity, potentially contributing to heightened systemic cytokine responses.

While our results show higher IL-1β and IL-18 production in CSU patients carrying the *NLRP3* rs10754558 C allele, direct mechanistic confirmation—including protein-level assessment (NLRP3, pro-IL-1β, pro-IL-18, caspase-1) and pharmacological inhibition (e.g., MCC950)—remains to be explored. Accordingly, these findings are described as associative rather than mechanistic, and further studies are warranted to clarify the functional role of NLRP3 in CSU.

IL-1β and IL-18 are key regulators of inflammatory processes and play essential roles in infection and cancer (43). n our study, LPS-stimulated whole blood cultures from CSU patients and controls showed higher IL-1β and IL-18 production in C/C carriers of *NLRP3* rs10754558 compared with C/G and G/G genotypes. Although Su et al. (2020) reported no genotype-dependent differences in systemic lupus erythematosus patients (32), this discrepancy may be attributable to the use of serum-based measurements rather than cell-based assays.

Overall and genotype-specific IL-1β and IL-18 production was significantly higher in CSU patients compared with controls, consistent with previous reports (7, 44), although some studies did not detect differences in serum cytokine levels (45–47). IL-1β and IL-18 are differentially regulated by inflammatory stimuli: IL-18 expression is sustained following TLR stimulation, whereas IL-1β induction is transient (43).

The observed genotype-dependent differences in clinical and laboratory parameters further support a pathogenic role for the NLRP3 inflammasome in CSU. Specifically, UAS7 scores were elevated in C/C and C/G carriers, total IgE and LPS-stimulated IL-1β were higher in carriers of at least one C allele (dominant model), and LPS-stimulated IL-1β and IL-18 were increased in C/C homozygotes (recessive model). Interestingly, C/C carriers in the recessive model exhibited lower UAS7 scores despite heightened cytokine production. This apparent discordance likely reflects the prospective and subjective nature of UAS7, as well as the potential initiation of therapy prior to assessment. These findings suggest that functional inflammasome readouts may capture immune activation not fully reflected by patient-reported outcomes. Overall, they indicate that heightened inflammasome activity contributes to CSU susceptibility and immune phenotype independently of conventional clinical severity metrics, highlighting the potential of targeting inflammasome pathways in genetically predisposed individuals.

Consistent with prior studies (7, 45, 46, 48), LPS-stimulated IL-1β and IL-18 production was not influenced by ASST reactivity or ESR, and cytokine levels did not correlate with age, BMI, UAS7, or total IgE. However, higher IL-1β and IL-6 mRNA expressions have been reported in skin biopsies from ASST-positive CSU patients (6), and IL-18 levels have been associated with disease severity (14, 45). IL-1β released by MCs promotes neutrophil migration and vascular leakage, implicating it in wheal formation via autocrine activation (6, 49), whereas IL-18 enhances Th1, Th2, and Th17 responses, MC degranulation, histamine release, IgE production, and recruitment of neutrophils and eosinophils (49, 50).

The UAS7 represents the gold standard for quantifying CSU activity, integrating wheal number and pruritus severity, and is typically assessed prospectively over seven consecutive days (50). In line with prior studies (51, 52), UAS7 scores in our cohort ranged from 10 to 42, with total IgE levels above the normal range; however, no significant correlation was observed between UAS7 and either BMI or IgE levels. These findings suggest that, although IL-1β/IL-18 production and NLRP3 polymorphism are associated with inflammatory responsiveness, traditional clinical parameters such as UAS7 may not fully capture CSU activity.

CSU is currently understood to encompass at least two major immunological endotypes: type I autoimmune (autoallergic) CSU, driven by autoreactive IgE antibodies, and type IIb autoimmune CSU, characterized by IgG and/or IgM autoantibodies targeting FcϵRIα or FcϵRIα-bound IgE (53). Although total serum IgE levels are often elevated in CSU, they vary widely among patients and do not consistently correlate with disease activity (54). In this context, ASST serves as an *in vivo* indicator of autoreactivity rather than a definitive marker of autoimmune disease. The high prevalence of ASST positivity observed in the present study (>70%) falls within the upper range reported in the literature (17) and supports the presence of mast cell–activating factors in a substantial subset of patients. Notably, ASST-positive patients exhibited higher UAS7 scores and total serum IgE levels, indicating a more active clinical phenotype, although ASST reactivity was not associated with ESR, consistent with prior reports (55).

## 5 Limitations

Despite providing novel insights into the role of *NLRP3* rs10754558 in CSU, this study has several limitations. First, the single-center, case–control design with a modest sample size may limit generalizability. Second, the analysis focused on a single NLRP3 polymorphism, and additional genetic variants or inflammasome components may contribute to disease heterogeneity. Third, functional assays were performed using whole blood cultures, which preserve physiological cell–cell interactions and provide an integrated view of systemic immune responses but do not allow precise attribution of cytokine production to specific immune cell subsets. LPS-stimulation alone delivers a priming signal and does not fully replicate canonical NLRP3 inflammasome activation, which typically requires a secondary stimulus such as ATP or nigericin; the absence of protein-level validation (e.g., pro–IL-1β, pro–IL-18, or caspase-1) further limits mechanistic interpretation. Fourth, cytokine measurements from whole blood may not accurately reflect tissue-level processes in the skin, the primary site of CSU pathology; tissue-based approaches, such as skin biopsy or *in situ* cytokine assessment, would provide more direct insight into local immune mechanisms. Fifth, some participants may have initiated therapy prior to assessment, potentially influencing cytokine responses and UAS7 scores. Finally, conventional clinical metrics, including UAS7 and total IgE, may not fully capture underlying immune dysregulation, as suggested by partial discordance with functional cytokine responses.

## 6 Conclusion

The present study demonstrates that the *NLRP3* rs10754558 polymorphism is associated with CSU susceptibility, with the C allele in particular linked to heightened IL-1β and IL-18 production in LPS-stimulated whole blood cultures. These genotype-dependent immune alterations underscore the contribution of inflammasome pathways to CSU susceptibility, independent of conventional clinical severity measures. While systemic assays provide integrated insights, future studies using dual-signal inflammasome activation and protein-level analyses are needed to clarify mechanistic pathways, and tissue-based approaches are warranted to investigate local inflammatory processes. Together, these studies could inform potential therapeutic avenues targeting the NLRP3 inflammasome in genetically predisposed individuals.

## Data Availability

The original contributions presented in the study are included in the article/**Supplementary Material**. Further inquiries can be directed to the corresponding author.

## References

[1] Kaplan A Lebwohl M Giménez-Arnau AM Hide M Armstrong AW Maurer M . Chronic spontaneous urticaria: Focus on pathophysiology to unlock treatment advances. Allergy. (2023) 78:389–401. doi: 10.1111/all.15603. PMID: 36448493

[2] Wang D Duncan B Li X Shi J . The role of Nlrp3 inflammasome in infection-related, immune-mediated and autoimmune skin diseases. J Dermatol Sci. (2020) 98:146–51. doi: 10.1016/j.jdermsci.2020.03.001. PMID: 32173167

[3] Haidar L Bănărescu CF Uţa C Zimbru EL Zimbru RI Tîrziu A . Beyond the skin: Exploring the gut-skin axis in chronic spontaneous urticaria and other inflammatory skin diseases. Biomedicines. (2025) 13. doi: 10.3390/biomedicines13082014. PMID: 40868265 PMC12383297

[4] Guo H Callaway JB Ting JPY . Inflammasomes: Mechanism of action, role in disease, and therapeutics. Nat Med. (2015) 21:677–87. doi: 10.1038/nm.3893. PMID: 26121197 PMC4519035

[5] Kawamura T Ogawa Y Aoki R Shimada S . Innate and intrinsic antiviral immunity in skin. J Dermatol Sci. (2014) 75:159–66. doi: 10.1016/j.jdermsci.2014.05.004. PMID: 24928148

[6] de Montjoye L Choteau M Herman A Hendrickx E Chéou P Baeck M . Il-6 and Il-1β expression is increased in autologous serum skin test of patients with chronic spontaneous urticaria. Allergy. (2019) 74:2522–4. doi: 10.1111/all.13928. PMID: 31125442

[7] Puxeddu I Italiani P Giungato P Pratesi F Panza F Bartaloni D . Free Il-18 and Il-33 cytokines in chronic spontaneous urticaria. Cytokine. (2013) 61:741–3. doi: 10.1016/j.cyto.2013.01.015. PMID: 23433789

[8] Wu Z Wu S Liang T . Association of Nlrp3 Rs35829419 and Rs10754558 polymorphisms with risks of autoimmune diseases: A systematic review and meta-analysis. Front Genet. (2021) 12:690860. doi: 10.3389/fgene.2021.690860. PMID: 34367252 PMC8340881

[9] KamadaAJ PontilloA GuimarãesRL LoureiroP CrovellaS BrandãoLAC . Nlrp3 polymorphism is associated with protection against human T-lymphotropic virus 1 infection. Memórias do Instituto Oswaldo Cruz. (2014) 109:957–60. doi: 10.1590/0074-0276140154. PMID: 25411003

[10] Borelli V Moura RR Trevisan E Crovella S . Nlrp1 and Nlrp3 polymorphisms in mesothelioma patients and asbestos exposed individuals a population-based autopsy study from North East Italy. Infect Agents Cancer. (2015) 10:26. doi: 10.1186/s13027-015-0022-0. PMID: 26236392 PMC4521353

[11] Mian SA Kulasekararaj AG Kizilors A McGraw KL Kordasti S Menzel S . Nlrp3 inflammosome polymorphisms are enriched in myelodysplastic syndrome patients with autoimmune disorders. Blood. (2015) 126:1659. doi: 10.1182/blood.V126.23.1659.1659

[12] Zhou D Wang X Chen T Wen W Liu Y Wu Y . The Nlrp3 Rs10754558 polymorphism is associated with the occurrence and prognosis of coronary artery disease in the Chinese Han population. BioMed Res Int. (2016) 2016:3185397. doi: 10.1155/2016/3185397. PMID: 27110561 PMC4823501

[13] El Gendy A Abo Ali FH Baioumy SA Taha SI El -Bassiouny M Abdel Latif OM . Nod-like receptor family pyrin domain containing 3 (Rs10754558) gene polymorphism in chronic spontaneous urticaria: A pilot case-control study. Immunobiology. (2025) 230:152868. doi: 10.1016/j.imbio.2025.152868. PMID: 39818117

[14] Abdel-Bary A Elnily D Soror O Youssef M . Serum interleukin-18 and immunoglobulin E in chronic spontaneous urticaria and their relation to severity of the disease. J Egyp Women’s Dermatol Soc. (2022) 19:88–93. doi: 10.4103/jewd.jewd_59_21. PMID: 41476358

[15] Zuberbier T Abdul Latiff AH Abuzakouk M Aquilina S Asero R Baker D . The international Eaaci/Ga²len/Euroguiderm/Apaaaci guideline for the definition, classification, diagnosis, and management of urticaria. Allergy. (2022) 77:734–66. doi: 10.1111/all.15090. PMID: 34536239

[16] Eknoyan G . Adolphe Quetelet (1796–1874)—the average man and indices of obesity. Nephrol Dialysis Transplant. (2007) 23:47–51. doi: 10.1093/ndt/gfm517. PMID: 17890752

[17] Konstantinou GN Asero R Maurer M Sabroe RA Schmid-Grendelmeier P Grattan CEH . Eaaci/Ga2len task force consensus report: The autologous serum skin test in urticaria. Allergy. (2009) 64:1256–68. doi: 10.1111/j.1398-9995.2009.02132.x. PMID: 19650847

[18] Rodas L Martínez S Riera-Sampol A Moir HJ Tauler P . Blood cell *in vitro* cytokine production in response to lipopolysaccharide stimulation in a healthy population: Effects of age, sex, and smoking. Cells. (2022) 11:103. doi: 10.3390/cells11010103. PMID: 35011664 PMC8750398

[19] Haque KA Pfeiffer RM Beerman MB Struewing JP Chanock SJ Bergen AW . Performance of high-throughput DNA quantification methods. BMC Biotech. (2003) 3:20. doi: 10.1186/1472-6750-3-20. PMID: 14583097 PMC280658

[20] Pinzón-Fernández MV Saavedra-Torres JS López Garzón NA Pachon-Bueno JS Tamayo-Giraldo FJ Rojas Gomez MC . Nlrp3 and beyond: Inflammasomes as central cellular hub and emerging therapeutic target in inflammation and disease. Front Immunol. (2025) 16:1624770. doi: 10.3389/fimmu.2025.1624770. PMID: 40959087 PMC12433879

[21] Beck LA Bernstein JA Maurer M . A review of international recommendations for the diagnosis and management of chronic urticaria. Acta Derm Venereol. (2017) 97:149–58. doi: 10.2340/00015555-2496. PMID: 27349620

[22] Gonçalo M Gimenéz-Arnau A Al-Ahmad M Ben-Shoshan M Bernstein JA Ensina LF . The global burden of chronic urticaria for the patient and society. Br J Dermatol. (2021) 184:226–36. doi: 10.1111/bjd.19561. PMID: 32956489

[23] Hoffman HM Rosengren S Boyle DL Cho JY Nayar J Mueller JL . Prevention of cold-associated acute inflammation in familial cold autoinflammatory syndrome by interleukin-1 receptor antagonist. Lancet. (2004) 364:1779–85. doi: 10.1016/s0140-6736(04)17401-1. PMID: 15541451 PMC4321997

[24] Nakamura Y Franchi L Kambe N Meng G Strober W Núñez G . Critical role for mast cells in interleukin-1β-driven skin inflammation associated with an activating mutation in the Nlrp3 protein. Immunity. (2012) 37:85–95. doi: 10.1016/j.immuni.2012.04.013. PMID: 22819042 PMC3411177

[25] Imani D Azimi A Salehi Z Rezaei N Emamnejad R Sadr M . Association of nod-like receptor protein-3 single nucleotide gene polymorphisms and expression with the susceptibility to relapsing–remitting multiple sclerosis. Int J Immunogenet. (2018) 45:329–36. doi: 10.1111/iji.12401. PMID: 30264444

[26] Jenko B Praprotnik S Tomšic M Dolžan V . Nlrp3 and Card8 polymorphisms influence higher disease activity in rheumatoid arthritis. J Med Biochem. (2016) 35:319–23. doi: 10.1515/jomb-2016-0008. PMID: 28356883 PMC5346810

[27] Addobbati C da Cruz HLA Adelino JE Melo Tavares Ramos AL Fragoso TS Domingues A . Polymorphisms and expression of inflammasome genes are associated with the development and severity of rheumatoid arthritis in Brazilian patients. Inflammation Res. (2018) 67:255–64. doi: 10.1007/s00011-017-1119-2. PMID: 29230505

[28] Juneblad K Kastbom A Johansson L Rantapää-Dahlqvist S Söderkvist P Alenius GM . Association between inflammasome-related polymorphisms and psoriatic arthritis. Scand J Rheumatol. (2021) 50:206–12. doi: 10.1080/03009742.2020.1834611. PMID: 33300400

[29] Pontillo A Vendramin A Catamo E Fabris A Crovella S . The missense variation Q705k in Cias1/Nalp3/Nlrp3 gene and an Nlrp1 haplotype are associated with celiac disease. Am J Gastroenterol. (2011) 106:539–44. doi: 10.1038/ajg.2010.474. PMID: 21245836

[30] Smigoc Schweiger D Goricar K Hovnik T Mendez A Bratina N Brecelj J . Dual role of Ptpn22 but not Nlrp3 inflammasome polymorphisms in type 1 diabetes and celiac disease in children. Front Pediatr. (2019) 7:63. doi: 10.3389/fped.2019.00063. PMID: 30915320 PMC6422865

[31] Agah E Nafissi S Saleh F Sarraf P Tafakhori A Mousavi SV . Investigating the possible association between Nlrp3 gene polymorphisms and myasthenia gravis. Muscle Nerve. (2021) 63:730–6. doi: 10.1002/mus.27193. PMID: 33533549

[32] Su Z Niu Q Huang Z Yang B Zhang J . Association of nucleotide-binding oligomerization domain-like receptor family pyrin domain-containing protein 3 polymorphisms with systemic lupus erythematosus disease activity and biomarker levels: A case-control study in Chinese population. Med Baltimore. (2020) 99:e21888. doi: 10.1097/md.0000000000021888. PMID: 32871918 PMC7458188

[33] Hitomi Y Ebisawa M Tomikawa M Imai T Komata T Hirota T . Associations of functional Nlrp3 polymorphisms with susceptibility to food-induced anaphylaxis and aspirin-induced asthma. J Allergy Clin Immunol. (2009) 124:779–785.e6. doi: 10.1016/j.jaci.2009.07.044. PMID: 19767079

[34] Lee YH Bae SC . Association between functional Nlrp3 polymorphisms and susceptibility to autoimmune and inflammatory diseases: A meta-analysis. Lupus. (2016) 25:1558–66. doi: 10.1177/0961203316644336. PMID: 27060062

[35] Pontillo A Brandão LA Guimarães RL Segat L Athanasakis E Crovella S . A 3′ Utr Snp in Nlrp3 gene is associated with susceptibility to Hiv-1 infection. JAIDS J Acq Immune Defic Syndromes. (2010) 54:236–40. doi: 10.1097/QAI.0b013e3181dd17d4. PMID: 20502346

[36] Paramel GV Sirsjö A Fransén K . Role of genetic alterations in the Nlrp3 and Card8 genes in health and disease. Mediators Inflammation. (2015) 2015:846782. doi: 10.1155/2015/846782. PMID: 25788762 PMC4348606

[37] Zhan X Li Q Xu G Xiao X Bai Z . The mechanism of Nlrp3 inflammasome activation and its pharmacological inhibitors. Front Immunol. (2022) 13:1109938. doi: 10.3389/fimmu.2022.1109938. PMID: 36741414 PMC9889537

[38] Ferrari D Chiozzi P Falzoni S Hanau S Di Virgilio F . Purinergic modulation of interleukin-1 beta release from microglial cells stimulated with bacterial endotoxin. J Exp Med. (1997) 185:579–82. doi: 10.1084/jem.185.3.579. PMID: 9053458 PMC2196027

[39] Netea MG Nold-Petry CA Nold MF Joosten LA Opitz B van der Meer JH . Differential requirement for the activation of the inflammasome for processing and release of Il-1beta in monocytes and macrophages. Blood. (2009) 113:2324–35. doi: 10.1182/blood-2008-03-146720. PMID: 19104081 PMC2652374

[40] Gattorno M Tassi S Carta S Delfino L Ferlito F Pelagatti MA . Pattern of interleukin-1beta secretion in response to lipopolysaccharide and Atp before and after interleukin-1 blockade in patients with Cias1 mutations. Arthritis Rheum. (2007) 56:3138–48. doi: 10.1002/art.22842. PMID: 17763411

[41] Goldbach-Mansky R Dailey NJ Canna SW Gelabert A Jones J Rubin BI . Neonatal-onset multisystem inflammatory disease responsive to interleukin-1beta inhibition. N Engl J Med. (2006) 355:581–92. doi: 10.1056/NEJMoa055137. PMID: 16899778 PMC4178954

[42] Drenth JP van Deuren M van der Ven-Jongekrijg J Schalkwijk CG van der Meer JW . Cytokine activation during attacks of the hyperimmunoglobulinemia D and periodic fever syndrome. Blood. (1995) 85:3586–93. doi: 10.1182/blood.V85.12.3586.bloodjournal85123586 7780142

[43] Zhu Q Kanneganti TD . Cutting edge: Distinct regulatory mechanisms control proinflammatory cytokines Il-18 and Il-1β. J Immunol. (2017) 198:4210–5. doi: 10.4049/jimmunol.1700352. PMID: 28468974 PMC5544497

[44] dos Santos JC Azor MH Nojima VY Lourenço FD Prearo E Maruta CW . Increased circulating pro-inflammatory cytokines and imbalanced regulatory T-cell cytokines production in chronic idiopathic urticaria. Int Immunopharmacol. (2008) 8:1433–40. doi: 10.1016/j.intimp.2008.05.016. PMID: 18586117

[45] Tedeschi A Lorini M Suli C Asero R . Serum interleukin‐18 in patients with chronic ordinary urticaria: Association with disease activity. Clin Exp Dermatol: Exp Dermatol. (2007) 32:568–70. doi: 10.1111/j.1365-2230.2007.02450.x. PMID: 17509061

[46] Rasool R Ashiq I Shera IA Yousuf Q Shah ZA . Study of serum interleukin (Il) 18 and Il-6 levels in relation with the clinical disease severity in chronic idiopathic urticaria patients of Kashmir (North India). Asia Pac Allergy. (2014) 4:206–11. doi: 10.5415/apallergy.2014.4.4.206. PMID: 25379480 PMC4215435

[47] Zheng R Qian L Yu J Li M Qian Q . Analysis of the changes in Th9 cells and related cytokines in the peripheral blood of spontaneous urticaria patients. BioMed Rep. (2017) 6:633–9. doi: 10.3892/br.2017.904. PMID: 28584634 PMC5449968

[48] Kurt E Aktas A Aksu K Keren M Dokumacioglu A Goss CH . Autologous serum skin test response in chronic spontaneous urticaria and respiratory diseases and its relationship with serum interleukin-18 level. Arch Dermatol Res. (2011) 303:643–9. doi: 10.1007/s00403-011-1144-x. PMID: 21448661

[49] Zhou B Li J Liu R Zhu L Peng C . The role of crosstalk of immune cells in pathogenesis of chronic spontaneous urticaria. Front Immunol. (2022) 13:879754. doi: 10.3389/fimmu.2022.879754. PMID: 35711438 PMC9193815

[50] Lee JH Cho DH Park HJ . Il-18 and cutaneous inflammatory diseases. Int J Mol Sci Internet. (2015) 16:29357–69. doi: 10.3390/ijms161226172 PMC4691115 26690141

[51] Nagayama K Watai K Sekiya K Iwata M Hashimoto Y Nakamura Y . Association between the severity of chronic spontaneous urticaria and sleep-disordered breathing. Allergol Int. (2022) 71:103–8. doi: 10.1016/j.alit.2021.08.001. PMID: 34511312

[52] Kulthanan K Nuchkull P Ungaksornpairote C Chularojanamontri L Tuchinda P . Prevalence and clinical correlation of serum immunoglobulin E in patients with chronic spontaneous urticaria. Ann Allergy Asthma Immunol. (2016) 116:258–259.e2. doi: 10.1016/j.anai.2015.12.007. PMID: 26803536

[53] Giménez-Arnau AM de Montjoye L Asero R Cugno M Kulthanan K Yanase Y . The pathogenesis of chronic spontaneous urticaria: The role of infiltrating cells. J Allergy Clin Immunol: In Pract. (2021) 9:2195–208. doi: 10.1016/j.jaip.2021.03.033. PMID: 33823316

[54] Altrichter S Fok JS Jiao Q Kolkhir P Pyatilova P Romero SM . Total Ige as a marker for chronic spontaneous urticaria. Allergy Asthma Immunol Res. (2021) 13:206–18. doi: 10.4168/aair.2021.13.2.206. PMID: 33474856 PMC7840871

[55] Ismail R Jamil A Md. Nor N Bakhtiar MF . A comparison of autologous serum, plasma, and whole blood for intradermal autoreactivity testing in patients with chronic spontaneous urticarial: A cross-sectional study. J Dermatol Dermatol Surg. (2022) 26:6–12. doi: 10.4103/jdds.jdds_72_21. PMID: 41480325

