# Supplementary material for: Association of NLRP3 rs10754558 polymorphism with inflammasome-related cytokine responses in chronic spontaneous urticaria
Source: Front Immunol. 2026 May 8;17:1804228. doi: 10.3389/fimmu.2026.1804228 (PMC13194015; doi:10.3389/fimmu.2026.1804228)
Supplement: Supplementary file 1 [file DataSheet1.pdf]

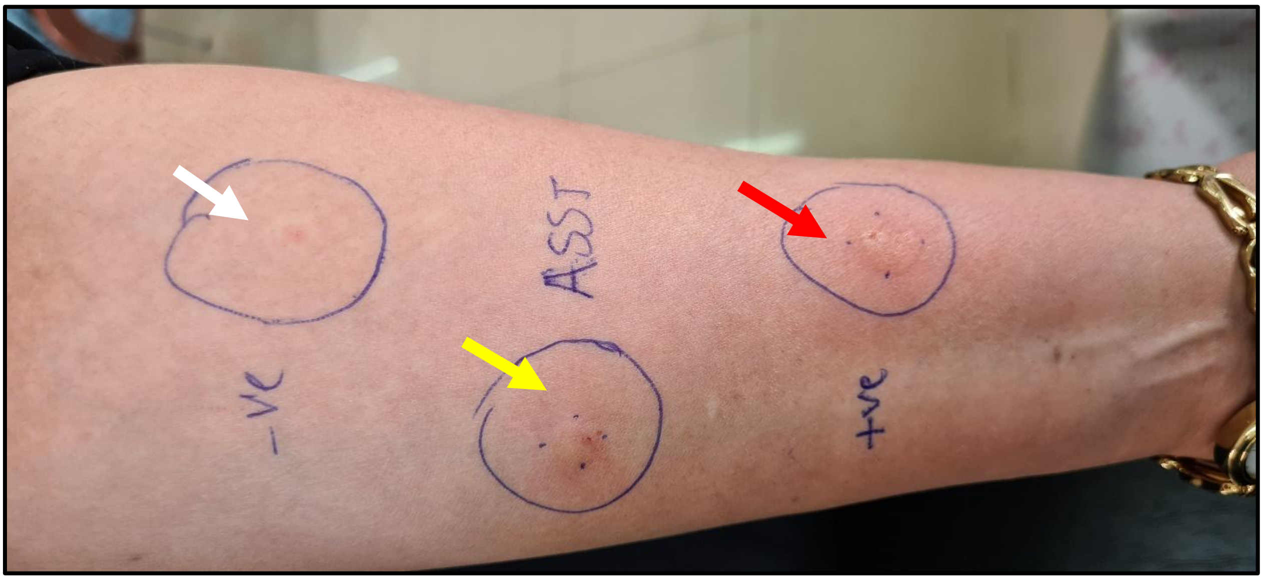

Supplementary Figure 1. Autologous serum skin test. Positive ASST elicited by intradermal injection of patient's undiluted autologous serum **(yellow arrow)** and normal saline, as negative control **(white arrow)**. **Red arrow** demonstrates positive control reaction using histamine (10 mg/mL) elicited by skin prick test.

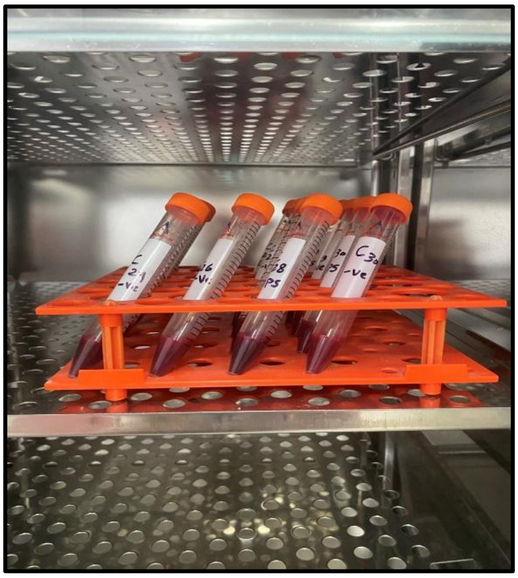

(A)

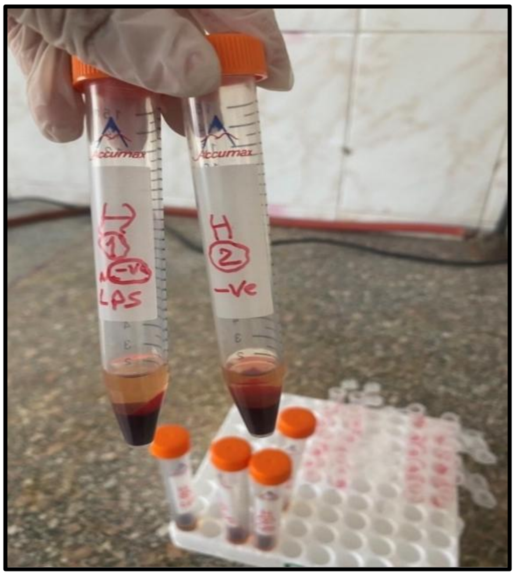

(B)

Supplementary Figure 2. (A) Whole blood cell cultures were incubated for 24 hours at 37˚C in humidified, 5% CO_2 (Carbon dioxide) atmosphere. **(B) Culture supernatant** after incubation period, were centrifuged at 1000×g for 10 min. Culture supernatants were harvested, collected, and stored at -20°C till use for cytokines' measurement.

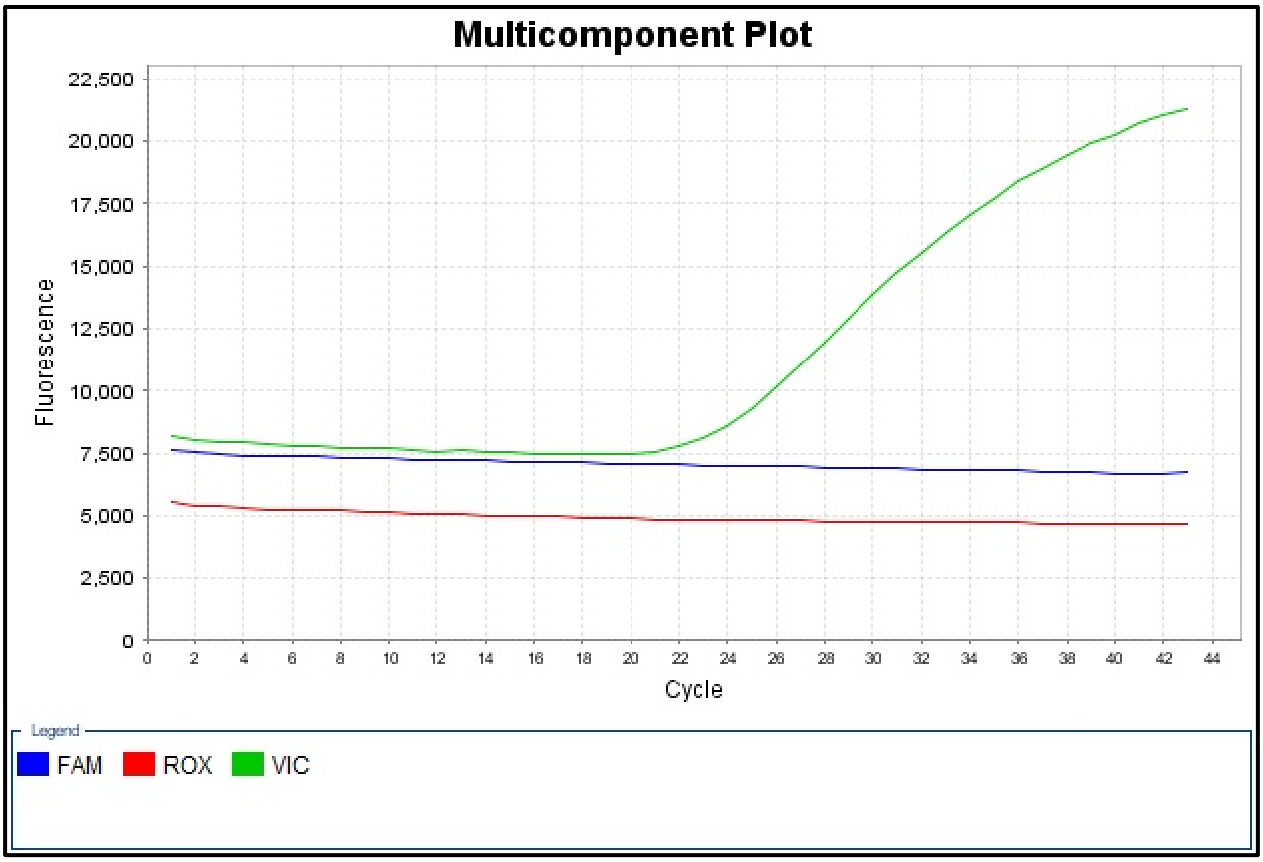

Supplementary Figure 3. NLRP3 rs10754558 polymorphism genotyping using Real-time PCR allelic discrimination assay. DNA was extracted from whole blood and subjected to qPCR analysis using specific Taqman^{TM} SNP genotyping assay for NLRP3 rs10754558 polymorphism genotyping. This plot shows VIC-dye **(green colored)** fluorescence only and hence **homozygosity for allele C.**

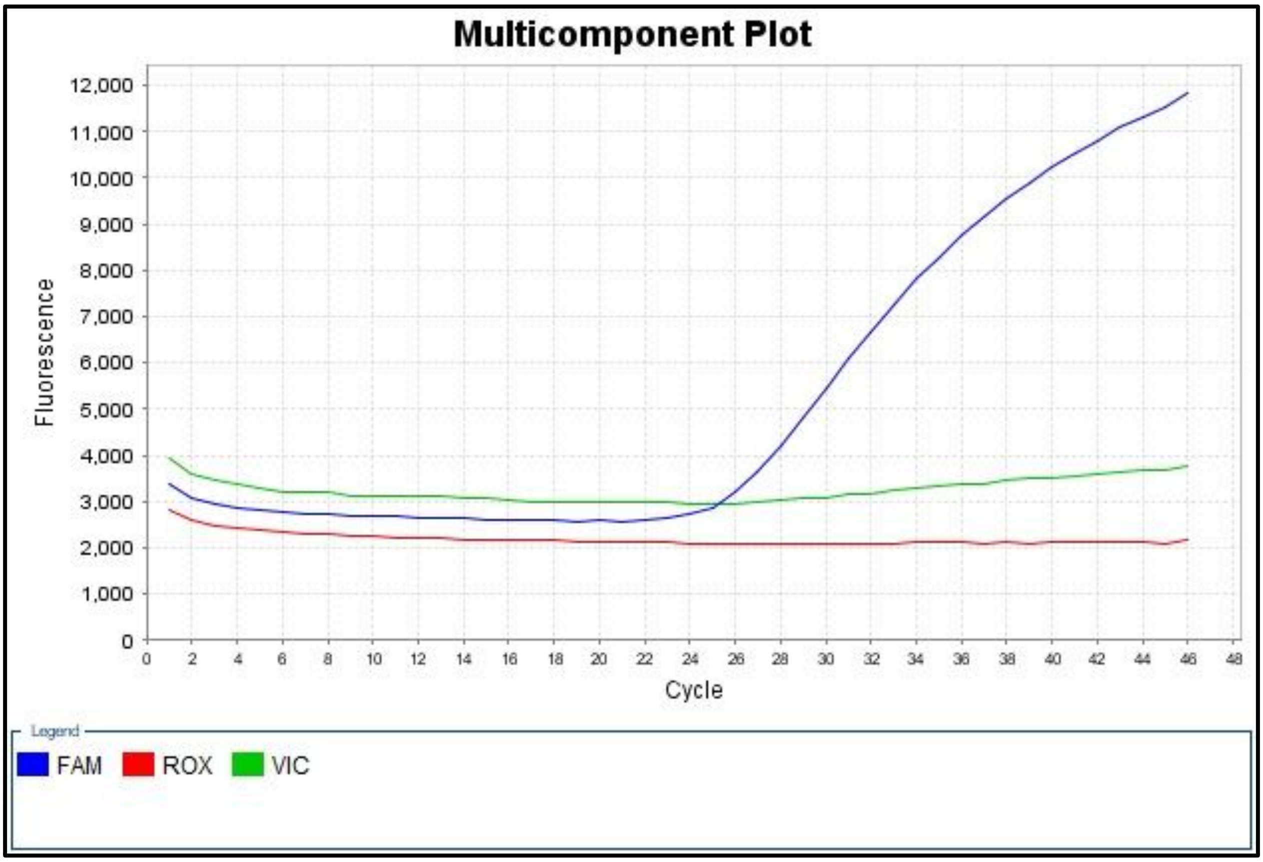

Supplementary Figure 4. NLRP3 rs10754558 polymorphism genotyping using Real-time PCR allelic discrimination assay. DNA was extracted from whole blood and subjected to qPCR analysis using specific Taqman^{TM} SNP genotyping assay for NLRP3 rs10754558 polymorphism genotyping. This plot shows **FAM-dye (blue colored)** fluorescence only and hence **homozygosity for allele G.**

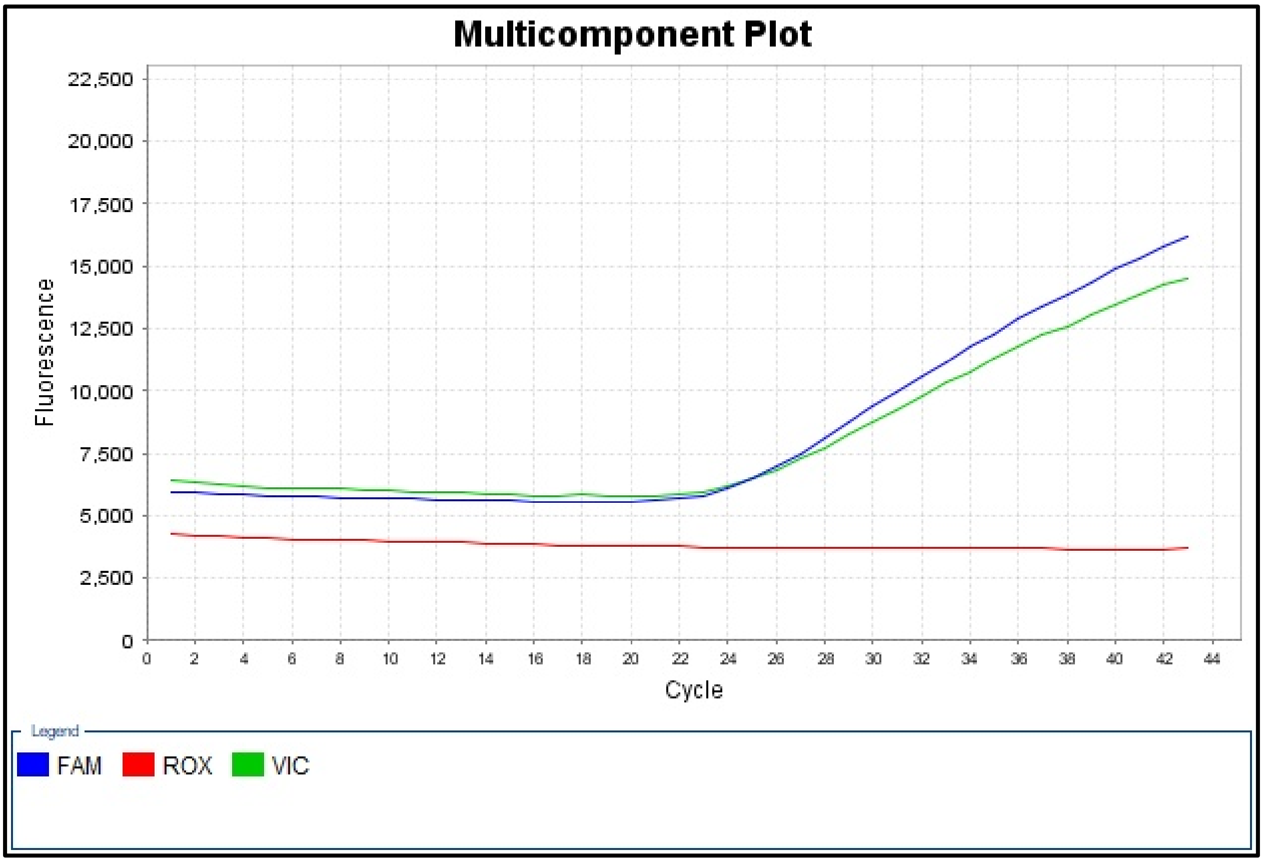

Supplementary Figure 5. NLRP3 rs10754558 polymorphism genotyping using Real-time PCR allelic discrimination assay. DNA was extracted from whole blood and subjected to qPCR analysis using specific TaqmanTM SNP genotyping assay for NLRP3 rs10754558 polymorphism genotyping. This plot shows both **VIC** **(green colored)** and **FAM** **(blue colored)** dye fluorescence and hence **C/G heterozygosity.**

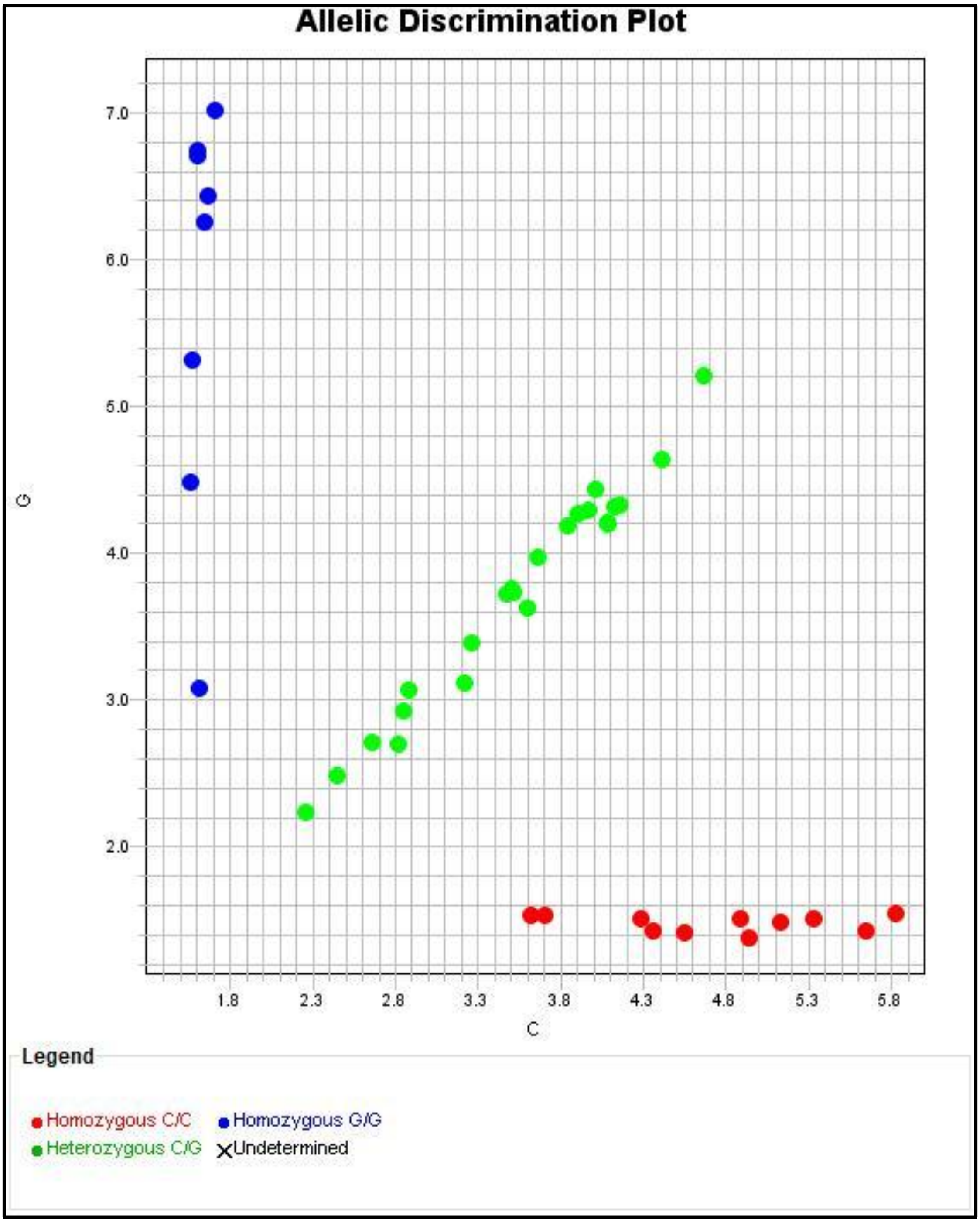

Supplementary Figure 6. NLRP3 rs10754558 allelic discrimination plot for CSU patients. **Red color** stands for **homozygous C/C** genotype, **green color** stands for heterozygous **C/G genotype** and **blue color** stands for **homozygous G/G genotype.**

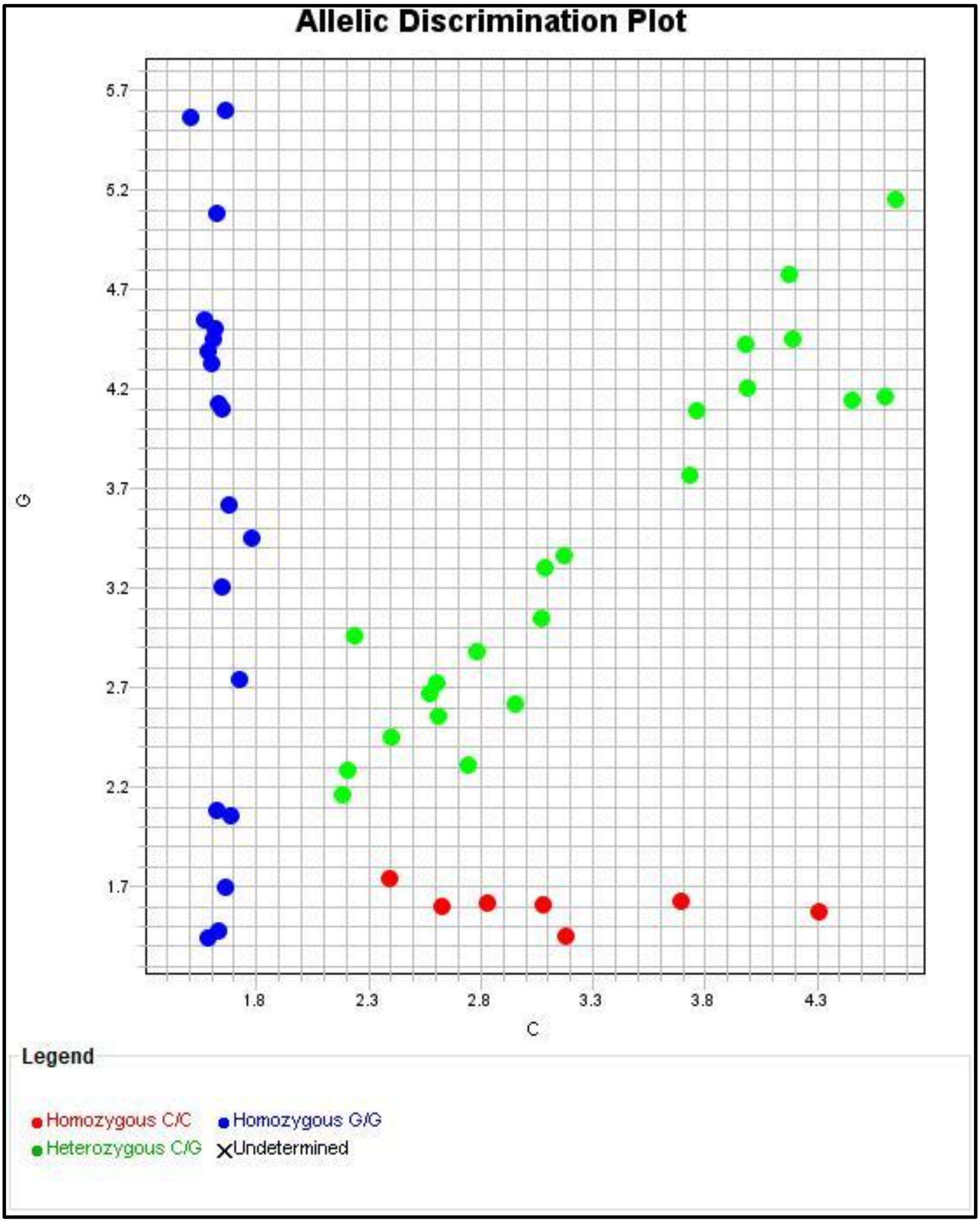

Supplementary Figure 7. NLRP3 rs10754558 allelic discrimination plot for healthy controls. **Red color** stands for **homozygous C/C** genotype, **green color** stands for heterozygous **C/G genotype** and **blue color** stands for **homozygous G/G genotype.**

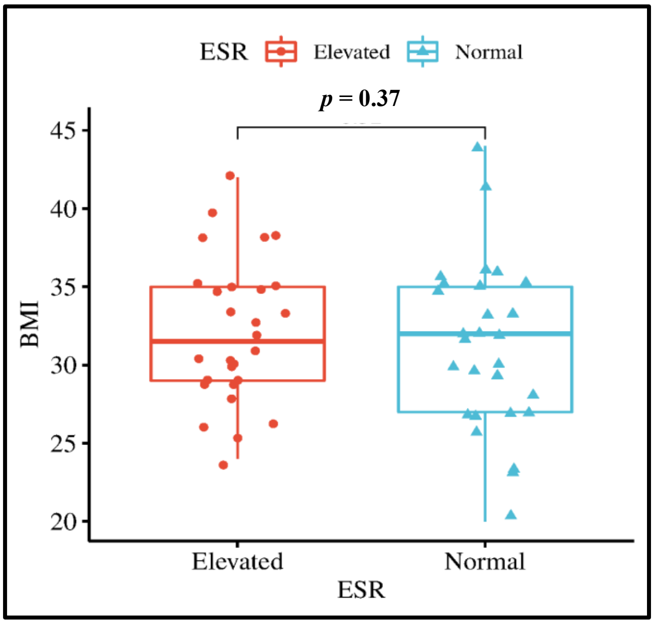

(A)

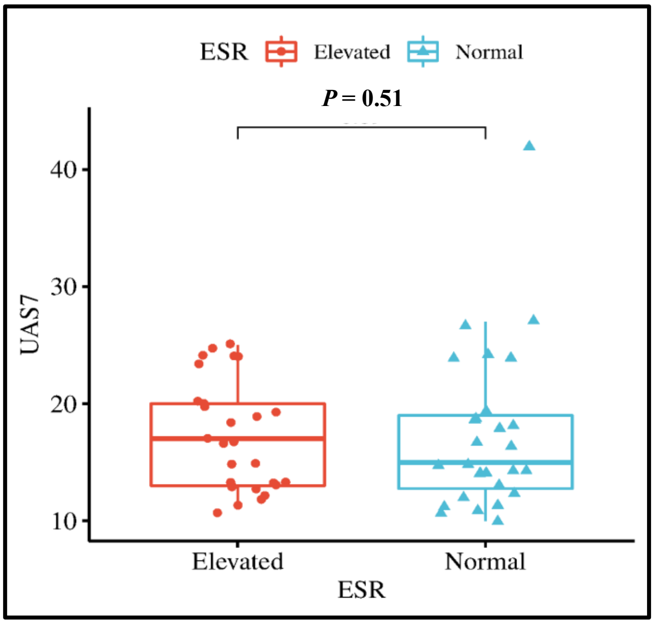

(B)

Supplementary Figure 8. Box plot showing (A) non- significant difference in BMI and **(B)** UAS7 between CSU patients with elevated and normal ESR (p = 0.37 and 0.51, respectively)**.**

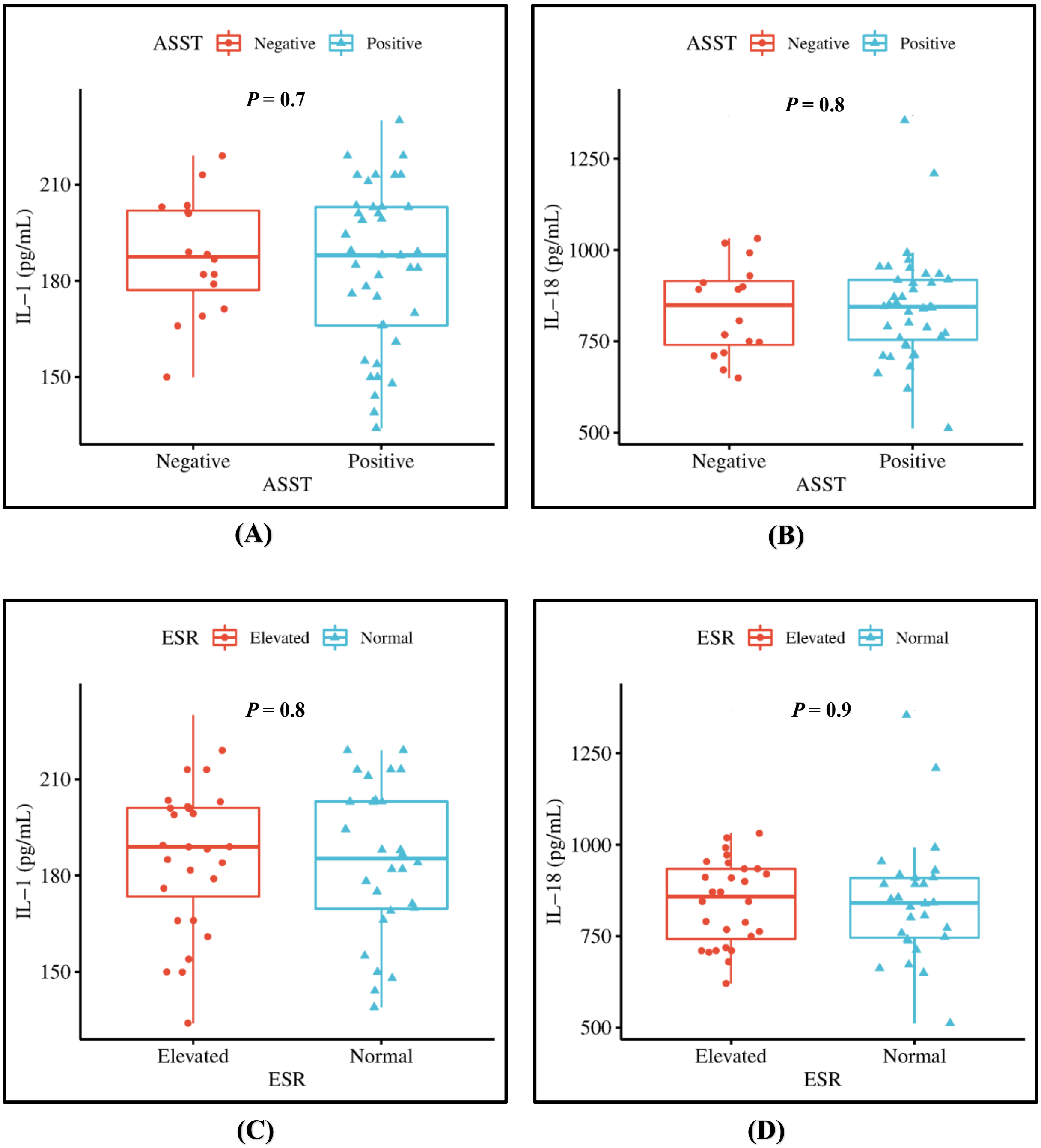

Supplementary Figure 9. **Box plots illustrating** non-significant difference between CSU patients with ASST negative and ASST positive in LPS induced **(A)** IL-1β production and **(B)** IL-18 (p = 0.7 and 0.8, respectively) and non-significant difference between patients with elevated and normal ESR in LPS induced **(C)** IL-1β production and **(D)** IL-18 as well 18 (p = 0.8 and 0.9, respectively).

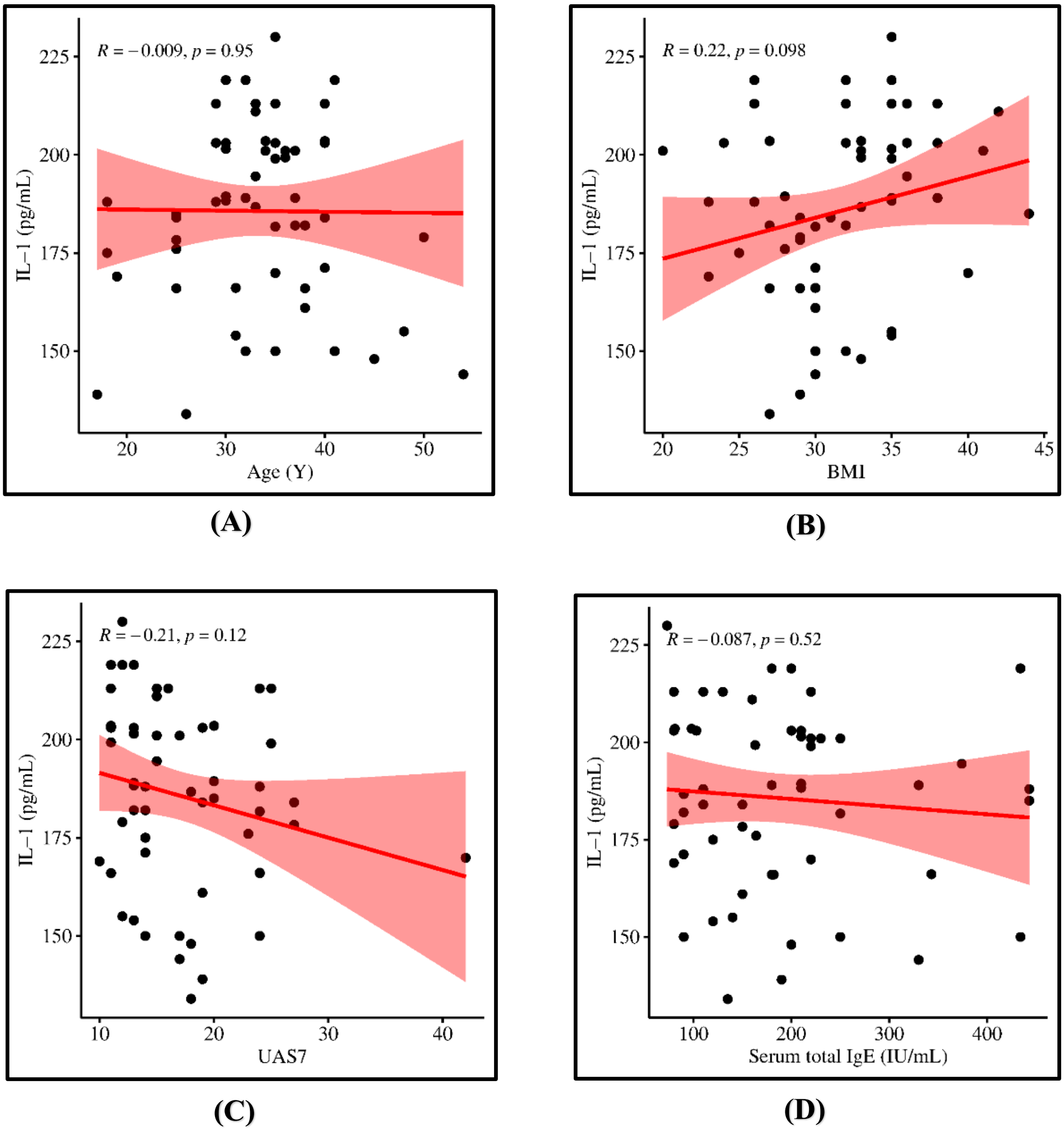

Supplementary Figure 10. Scatter plots showing non-significant correlations between IL-1β production induced by LPS and **(A)** age, **(B)** BMI, **(C)** UAS7, and **(D)** total IgE serum in CSU patients18 (p = 0.95, 0.098, 0.12, and 0.52 respectively).

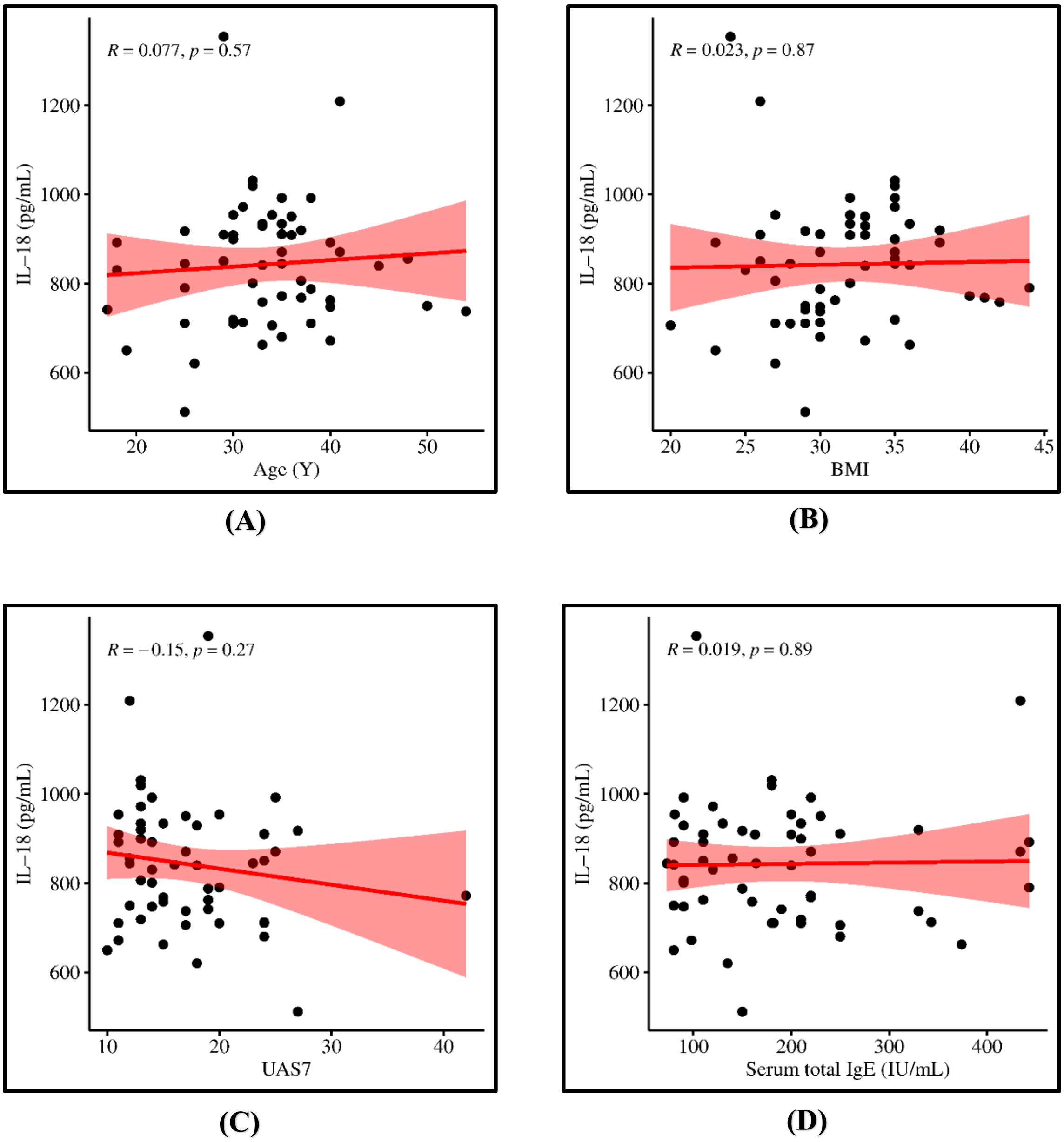

Supplementary Figure 11. Scatter plots showing non-significant correlations between IL-18 production induced by LPS and **(A)** age, **(B)** BMI, **(C)** UAS7, and **(D)** total IgE serum in CSU patients.
